# Microdissected Pyramidal Cell Proteomics of Alzheimer Brain Reveals Alterations in Creatine Kinase B-Type, 14-3-3-γ, and Heat Shock Cognate 71

**DOI:** 10.3389/fnagi.2021.735334

**Published:** 2021-11-19

**Authors:** Anna Sandebring-Matton, Michael Axenhus, Nenad Bogdanovic, Bengt Winblad, Sophia Schedin-Weiss, Per Nilsson, Lars O. Tjernberg

**Affiliations:** ^1^Division of Neurogeriatrics, Department of Neurobiology, Care Sciences and Society, Center for Alzheimer Research, Karolinska Institutet, Stockholm, Sweden; ^2^Division of Clinical Geriatrics, Department of Neurobiology, Care Sciences and Society, Center for Alzheimer Research, Karolinska Institutet, Stockholm, Sweden; ^3^Ageing Epidemiology (AGE) Research Unit, School of Public Health, Imperial College London, London, United Kingdom; ^4^Theme Inflammation and Aging, Karolinska University Hospital, Huddinge, Sweden; ^5^Clinical Chemistry, Karolinska University Hospital, Solna, Sweden

**Keywords:** Alzheimer’s disease, 14-3-3-γ, creatine kinase B, heat shock cognate 71, laser capture microscopy, proteomics, hippocampus

## Abstract

Novel insights on proteins involved in Alzheimer’s disease (AD) are needed. Since multiple cell types and matrix components are altered in AD, bulk analysis of brain tissue maybe difficult to interpret. In the current study, we isolated pyramidal cells from the cornu ammonis 1 (CA1) region of the hippocampus from five AD and five neurologically healthy donors using laser capture microdissection (LCM). The samples were analyzed by proteomics using ^18^O-labeled internal standard and nano-high-performance liquid chromatography coupled to tandem mass spectrometry (HPLC-MS/MS) for relative quantification. Fold change between AD and control was calculated for the proteins that were identified in at least two individual proteomes from each group. From the 10 cases analyzed, 62 proteins were identified in at least two AD cases and two control cases. Creatine kinase B-type (CKB), 14-3-3-γ, and heat shock cognate 71 (Hsc71), which have not been extensively studied in the context of the human AD brain previously, were selected for further studies by immunohistochemistry (IHC). In hippocampus, semi-quantitative measures of IHC staining of the three proteins confirmed the findings from our proteomic analysis. Studies of the same proteins in the frontal cortex revealed that the alterations remained for CKB and 14-3-3-γ but not for Hsc71. Protein upregulation in CA1 neurons of final stage AD is either a result of detrimental, pathological effects, or from cell-specific protective response mechanisms in surviving neurons. Based on previous findings from experimental studies, CKB and Hsc71 likely exhibit protective effects, whereas 14-3-3-γ may represent a detrimental pathway. These new players could reflect pathways of importance for the development of new therapeutic strategies.

## Introduction

Alzheimer’s disease (AD) is one of the most widespread public health issues in the world with around 50 million affected patients globally, and the number is expected to grow up to 75 million by the year 2030 (World Alzheimer Report, 2015). The cost of care for AD and various associated dementias is estimated at the US $818 billion globally, and the disease has a lot of strain on patients, caretakers, and society as a whole (Wimo et al., 2017). Past and current pharmaceutical trials aimed at finding an effective treatment for AD have a high failure rate, and the treatment strategies are often extraordinarily expensive. Pharmaceutical treatments in trials today mostly target known neuropathological hallmarks in AD (Cummings et al., 2014). One such element is the amyloid β-peptide (Aβ), which is generated physiologically by proteolytic cleavage of the amyloid precursor protein (APP) by the enzymes β- and γ-secretase (De-Paula et al., 2012). Together with neurofibrillary tangles (NFT) composed of hyperphosphorylated Tau protein, plaques composed of fibrillar Aβ is the major neuropathological hallmark of AD (Mott and Hulette, 2005). Although both Aβ and Tau evidently have major roles in AD, the fact remains that AD is a multifactorial disease and its complexity maybe due to additional, unknown key players.

Although AD is largely regarded as a gray matter disease, different types of brain cells are affected and are further accompanied by extracellular changes (Mathys et al., 2019). Homogenates of AD brain samples hence naturally include potential changes originating in glial cells, vascular cells, blood cells, and matrix components in addition to altered numbers and contents of neurons, making bulk analysis difficult to interpret (De Marchi et al., 2016; Xu et al., 2019). Hippocampal cornu ammonis 1 (CA1) and CA3 are areas rich in pyramidal neurons, mainly involved in memory formation, and severely affected in AD as reflected in models for AD (Kuczynski et al., 2008; Kuehn, 2020). Thus, enriching pyramidal cells by laser capture microdissection (LCM) is a powerful approach that allows a targeted downstream analysis. We have previously applied LCM to study both intra-neuronal Aβ in isolated hippocampal neurons from post-mortem AD brain and the combined proteome of isolated hippocampal pyramidal neurons pooled from six AD and six neurologically healthy cases, respectively (Aoki et al., 2008; Hashimoto et al., 2012a, b). Several proteins identified in the latter study have been investigated as biomarkers and pathological mediators in AD (Hashimoto M. et al., 2019; Hashimoto S. et al., 2019).

Due to advances in the sensitivity of proteomic analysis, the aim of the current study was to employ LCM and analyze each case separately by proteomics. Microdissection of 1,000 pyramidal CA1 neurons was performed in each of 10 brain donors (five AD cases and five controls), and unbiased proteomic analysis of each donor was performed using high-performance liquid chromatography coupled to tandem mass spectrometry (HPLC-MS/MS). From this analysis, three proteins with altered levels between groups, were selected for further analysis by immunohistochemistry (IHC); creatine kinase B-type (CKB), 14-3-3-γ, and heat shock cognate 71 (Hsc71).

CKB, which has both a cytosolic and mitochondrial form, is responsible for the transformation of adenosine triphosphate (ATP) to adenosine diphosphate (ADP) and thus maintains ATP reserves through phosphocreatine production. Previous studies in AD models have demonstrated that CKB may play an important role in the protection against Aβ-induced mitochondrial dysfunction (Bürklen et al., 2006; Hiramatsu et al., 2020a).

14-3-3-γ belongs to a highly conserved protein family mainly expressed in the brain where it regulates diverse functions by binding to kinases, signaling proteins, hydroxylases, and about 170 other ligands (Umahara et al., 2012). Epitopes of 14-3-3 are found in neuropathological protein aggregates in various neurological diseases, e.g., AD, Huntington’s disease, and Parkinson’s disease. Its main clinical context is, however, in Creutzfeldt Jakob’s disease (CJD) where it serves as a potent diagnostic marker for the particular degenerative changes (Muayqil et al., 2012). Yet it is unclear whether the 14-3-3-γ isoform is a pathological component of neurodegeneration or represents a compensatory or stress-reactive machinery based on neuronal protection (Vincent et al., 2001; Agarwal-Mawal et al., 2003; Sadik et al., 2009). 14-3-3-γ has been implicated to both counteract Aβ-mediated toxicity and have a neuronal protective effect via upregulation of astrocytic expression during ischemic conditions (Nelson and Alkon, 2007; Dong et al., 2010). Studies in AD have shown conflicting results with either decreased levels of 14-3-3-γ (Smani et al., 2018; Gu et al., 2020) or high protein abundance, such as interaction with NFT in AD (Layfield et al., 1996; Fountoulakis et al., 1999). 14-3-3-γ deficient mice demonstrate no increased mortality or behavior changes (Steinacker et al., 2005).

Neurodegenerative disorders are characterized by protein misfolding involving proteasomal- and autophagic dysfunction (Gandhi et al., 2019). Subsequently, there is a crucial need for chaperone activity. Hsc71 is a member of the Hsp70 family, which is composed of 17 different chaperones that are mainly involved in ATP-dependent protein folding and are believed to have neuroprotective effects (Pratt et al., 2015; Repalli and Meruelo, 2015). The functions by which Hsp70 proteins protect against neuronal damage are speculated to stem from either direct binding to Aβ42, disruption of Aβ metabolism, or conformation changes preventing Aβ42 neurotoxic accumulation and modulating Tau degradation (Kumar et al., 2007; Sarkar et al., 2008; Rivera et al., 2018; Wang et al., 2021) suggesting that this protein family is chaperones with promising neuroprotective properties in AD. Experimental studies in drosophila and mice support this notion since Hsp70 chaperones have been shown to protect against intracellular amyloid by interfering with the secretory pathways of APP (De Mena et al., 2017; Sun et al., 2017; Rezvykh et al., 2018).

Although these three proteins have previously been studied in the context of AD, their distribution patterns in in human brain have not been extensively studied to our knowledge. Thus quantitative and qualitative assessments of CKB, 14-3-3-γ, and Hsc71 in pyramidal cells and pyramidal rich regions of the hippocampal CA1 and CA3 and the frontal cortex were performed.

## Materials and Methods

### Human Brain Tissue

Human brain samples of the hippocampus and frontal cortex of AD and control were obtained from the Netherlands brain bank (NBB), Netherlands Institute for Neuroscience, Amsterdam.

Table 1 displays information about the clinical diagnosis, age of death, gender distribution, post-mortem processing intervals (PMI), APOE genotype, Braak staging, and amyloid load of the subjects. For LCM followed by proteomic analysis, fresh frozen hippocampal tissue was analyzed. Immunohistochemical analysis was performed in samples from other donors on fixed, paraffinized brain tissue. A majority of the hippocampal tissue blocks used in this study were from the middle part of the hippocampus.

**TABLE 1:** Demographics and clinical information of study subjects.

Methodology:		LCM and proteomics		Immunohistochemistry, hippocampus		Immunohistochemistry, frontal cortex	
			
Clinical diagnosis:		AD	Non-demented	AD	Non-demented	AD	Non-demented

Number of cases		(*n* = 5)	(*n* = 5)	(*n* = 5)	(*n* = 5)	(*n* = 5)	(*n* = 5)
Age of death (y), mean, SD (range)		78 ± 6.7 (70–88)	79 ± 7.1 (70–90)	84 ± 3.8 (78–89)	85 ± 6.5 (76–93)	85 ± 2.2 (83–89)	81 ± 6.4 (74–93)
Number (%) of males		2 (40%)	2 (40%)	2 (40%)	2 (40%)	2 (40%)	2 (40%)
Post-mortem interval (h), mean, SD (range)		6.0 ± 1.3 (5–8.3)	6.7 ± 2.2 (5–10.5)	4.2 ± 0.7 (3–5)	6.4 ± 0.9 (5–7.7)	6.2 ± 2.3 (5–7.5)	6.2 ± 2.3 (5–7.5)
ApoE genotype	Unknown	2			2		2
	3/4	1		4		4	
	3/3	2	4	1	3	1	3
	3/2		1				
Braak stage	0				1		
	I/II		5		3		5
	III/IV	1			1	1	
	V/VI	4		5		4	
Amyloid pathology	0		2		2		3
	A		3		3		2
	B						
	C	5		5		5	

*Descriptive data on the analyzed cases for each method and brain region, respectively. LCM, laser capture microdissection; AD, Alzheimer’s disease; ApoE, apolipoprotein E.*

### Laser Capture Microdissection and Protein Extraction

**FIGURE 1:**
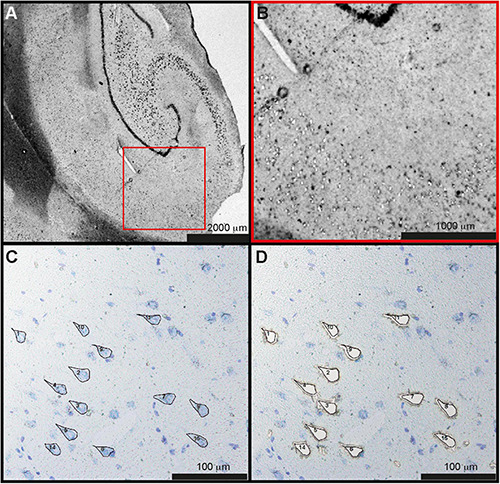
Laser capture microdissection of CA1 neurons. **(A)** Overview of the hippocampal section from one control case. **(B)** Magnified part of CA1 with individual, pyramidal cells microdissected from the tissue. **(C)** Pyramidal cells stained by toluidine blue were marked manually and **(D)** microdissected. A total of 1,000 neurons were dissected from each subject. CA1, cornu ammonis 1.

Fresh frozen tissue was exposed to brief staining and washing steps prior to dissection to keep the protein contents as intact as possible. Hippocampal sections (10 μm) were mounted on polyethylene terephthalate (PET)—membrane 1.4 μm slides (Leica Microsystems CMS GmbH, Wetzlar, Germany). The sections were fast fixed in 70% ethanol for 30 s, followed by three quick dips in distilled water (dH_2_O). To identify specific regions of the hippocampus, we subsequently stained the sections for 30 s in 0.5% toluidine blue (Sigma-Aldrich, St. Louis, MO, United States) dissolved in sodium acetate buffer, followed by three quick dips in dH_2_O and a 30-s wash in fresh dH_2_O. The sections were quickly dried in cold air and kept at +4°C until LCM. The steps for isolating pyramidal cells using a Leica microscope (LMD6000, Leica Microsystems) are shown in Figure 1. The dissected neurons were captured in 30 μl autoclaved water applied in the cap of low-binding tubes (Axygen, Union City, CA, United States). After the dissection of 1,000 CA1 pyramidal cells from each subject (originating from 3 to 5 different brain sections per case), tubes were centrifuged briefly in a mini desk-top centrifuge (VWR, Radnor, PA, United States) to collect the sample at the bottom of the tube. All samples were kept at −80°C until further sample preparation and analysis.

### Sample Preparation for Proteomics

The microdissected neurons were digested with trypsin (Promega Biotech, Stockholm, Sweden) (1:50 trypsin:protein ratio) in 100 mM ammonium bicarbonate (eluent for LC-MS, Sigma-Aldrich), 2 mM calcium chloride (anhydrous, free-flowing, Redi-Dri^3^ 97%, Sigma-Aldrich), 0.2% RapiGest (Waters Corporation, St Croix Falls, WI, United States) for 12 h in an incubator set at 37°C. The reaction was acidified by the addition of 1.5 μl 37% hydrogen chloride (Sigma-Aldrich) to hydrolyze RapiGest and centrifuged for 10 min at 15,000 rpm at +4°C (Eppendorf, Centrifuge 5417C, rotor FA45) to separate hydrophobic debris from hydrolyzed RapiGest and lipids. An internal ^18^O-labeled protein standard was prepared from pooling equal sized pieces of microdissected CA1 region from all individual samples which were then dried by speedvac (MAXI dry lyo) and digested with trypsin in the presence of ^18^O-labeled water (97 atom% ^18^O, Sigma-Aldrich) reconstituted in ammonium bicarbonate, calcium chloride, RapiGest and trypsin in H_2_^18^O.

The protein amount in the dissected brain cells was estimated by calculating the dissected tissue mass and appreciating a 10% protein content. Approximately 0.4 μg of tryptic peptides from each individual LCM sample of microdissected neurons was dissolved in 7.5 μl of 0.2% formic acid (99%, for LC/MS; VWR, Radnor, PA, United States) and sonicated for 5 min in an ice-cold water bath. Then, an equal amount of ^18^O-labeled internal standard was added to each sample to allow a relative quantification. Each sample + standard mixture was subsequently purified by ZipTipC_18_ chromatography (Millipore, Burlington, MA, United States) according to the instructions of manufacturer. The samples were dried by speedvac and stored at −80°C until LC-MS/MS analyses.

### Liquid Chromatography Coupled to Tandem Mass Spectrometry Analyses

The samples were dissolved in 0.2% formic acid, and a volume of 9 μl was injected to the LC-MS/MS (EASY-nLC 1000—Q Exactive, Thermo Fisher Scientific, Waltham, MA, United States). Mobile phase A was 0.1% formic acid and mobile phase B was acetonitrile, 0.1% formic acid. The peptides were eluted by a 120 min long gradient; 0-100 min 30% B, 100–120 min, 65% B, at a flow rate of 300 nl/min. The analytical column was a NANO-HPLC capillary C18 column, 0.075 × 150 mm (Nikkyo Technos, Tokyo, Japan), and the trap column was an Acclaim PepMap100 pre-column, 100 μm × 2 cm (Thermo Fisher Scientific, Waltham, MA, United States). The instrument settings were the following: Ionization: ESI; Capillary temp.: 275c; Spray voltage: 2.3 kV; type of fragmentation: HCD; resolution MS: 70,000; resolution MS2: 17,500; internal calibration method: lock mass m/z 371.10124, 445.12003 (Polysiloxane); isolation window: 2.0 m/z; AGC target: 1e5; maximum injection time: 60 ms; normalized collision energy (NCE): 28. Peptide selection was set at *m/z* 350–1,800 and 5% false discovery rate (FDR) was used as criteria.

The MS/MS data were searched with Proteome Discoverer (Ver. 1.4) with the MASCOT search engine (Ver. 2.5.1) against SwissProt database 2015_04 with search criteria; trypsin: max 1 missed cleavage site; taxonomy: homo sapiens; dynamic modifications: oxidation (M), label 18O (1 or 2) C-term [^18^O incomplete labeling (heavy + medium)/light)].

### Statistical Analyses of Proteins Identified by Liquid Chromatography Coupled to Tandem Mass Spectrometry

Protein abundance ratios and SDs were calculated for all proteins containing at least one unique peptide. This was performed by dividing the MS peak heights from ^18^O-labeled peptides with those from unlabeled peptides of each sample. To compensate for potential variations introduced during sample preparation, the protein ratios were—for each sample—normalized to the median (i.e., the number of proteins with ratio (AD/Ctr) higher than one were equal to the number of proteins with a ratio lower than one). For complete lists of detected proteins see Supplementary Table 1 and per case with peptide information see Supplementary Table 2. The abundance of proteins that were detected in two or more cases of the control and AD group, respectively, were each compared by student’s *t*-test (list sorted by *p*-value in Table 2). These 62 proteins were analyzed by principal component analysis (PCA) to identify overall proteomic differences between AD and controls. The PCA analysis was accomplished with Qlucore Omics Explorer version 3.3 (Qlucore, Lund, Sweden) and performed after filtering with a threshold of 1.05-fold change upon performing a two-group comparison between the control and AD. Missing values were adjusted for using the k-nearest neighbors’ algorithm, and the *p*-value was set to 0.05.

**TABLE 2:** Proteins detected by LCM-proteomics in at least two subjects from each group (AD and control).

62 proteins			Average STD/AD	Average STD/C	Ratio
					
ID	Protein	P	AD	C	AD/C
**P12277**	**Creatine kinase B-type**	**0.004**	**1.38**	**2.30**	**1.67**
Q13813	Spectrin alpha chain, non-erythrocytic 1	0.010	1.05	1.72	1.63
P14136	Glial fibrillary acidic protein	0.051	2.17	3.56	1.64
P07196	Neurofilament light polypeptide	0.104	0.53	1.33	2.52
Q01082	Spectrin beta chain, non-erythrocytic 1	0.116	1.58	0.86	0.54
Q16143	Beta-synuclein	0.136	1.72	0.53	0.31
P11137	Microtubule-associated protein 2	0.171	1.06	0.38	0.35
P40925	Malate dehydrogenase, cytoplasmic	0.181	0.55	1.60	2.91
P35908	Keratin, type II cytoskeletal 2 epidermal (contamination)	0.189	0.22	1.50	6.93
**P11142**	**Heat shock cognate 71** **kDa protein**	**0.192**	**0.72**	**1.53**	**2.11**
P13645	Keratin, type I cytoskeletal 10 (contamination)	0.236	0.53	1.07	2.02
**P61981**	**14-3-3 protein gamma**	**0.240**	**0.62**	**1.86**	**3.01**
P09936	Ubiquitin carboxyl-terminal hydrolase isozyme L1	0.254	2.26	1.73	0.77
Q16720	Plasma membrane calcium-transporting ATPase 3	0.264	2.48	1.36	0.55
P00338	L-lactate dehydrogenase A chain	0.274	0.29	0.75	2.60
Q9UQM7	Ca/calmodulin-dep, protein kinase type II subunit alpha	0.295	1.54	1.11	0.72
P07900	Heat shock protein HSP 90-alpha	0.305	1.63	1.09	0.67
P22626	Heterogeneous nuclear ribonucleoproteins A2/B1	0.328	0.61	1.24	2.04
P19367	Hexokinase-1	0.332	1.19	0.97	0.82
Q13423	NAD(P) transhydrogenase, mitochondrial	0.355	1.64	0.78	0.48
O43301	Heat shock 70 kDa protein 12A	0.358	0.53	2.62	4.97
P06576	ATP synthase subunit beta, mitochondrial	0.364	3.51	1.89	0.54
P17600	Synapsin-1	0.371	0.93	0.72	0.77
P05129	Protein kinase C gamma type	0.373	0.69	0.79	1.15
P68366	Tubulin alpha-4A chain	0.379	0.86	1.27	1.47
P10809	60 kDa heat shock protein, mitochondrial	0.389	5.47	1.41	0.26
P38606	V-type proton ATPase catalytic subunit A	0.407	0.82	1.14	1.38
P00505	Aspartate aminotransferase, mitochondrial	0.418	0.77	1.90	2.47
P04350	Tubulin beta-4A chain	0.423	1.54	2.11	1.37
P68104	Elongation factor 1-alpha 1	0.450	0.55	1.08	1.96
Q13885	Tubulin beta-2A chain	0.464	2.00	1.25	0.62
P10636	Microtubule-associated protein tau	0.469	1.26	0.98	0.78
P63104	14-3-3 protein zeta/delta	0.471	1.14	1.50	1.31
P46459	Vesicle-fusing ATPase	0.476	1.46	1.26	0.87
P27348	14-3-3 protein theta	0.488	1.84	1.24	0.68
P69905	Hemoglobin subunit alpha	0.495	1.21	0.99	0.81
P06744	Glucose-6-phosphate isomerase	0.496	1.47	1.14	0.77
P0DP23	Calmodulin	0.530	4.27	1.78	0.42
P13637	Sodium/potassium-transporting ATPase subunit alpha-3	0.553	3.08	1.91	0.62
P02686	Myelin basic protein	0.553	2.25	1.76	0.78
P07195	L-lactate dehydrogenase B chain	0.575	1.29	2.31	1.80
P25705	ATP synthase subunit alpha, mitochondrial	0.618	0.89	1.03	1.15
P60880	Synaptosomal-associated protein 25	0.641	1.36	1.54	1.13
P60201	Myelin proteolipid protein	0.666	1.78	1.54	0.86
Q6NXT2	Histone H3.3C	0.667	0.61	0.68	1.11
P23246	Splicing factor, proline- and glutamine-rich	0.709	0.49	0.66	1.34
P07339	Cathepsin D	0.719	1.21	0.95	0.79
P14618	Pyruvate kinase isozymes M1/M2	0.726	1.54	1.41	0.92
P21281	V-type proton ATPase subunit B, brain isoform	0.740	1.08	0.92	0.84
P60709	Actin, cytoplasmic 1	0.745	2.33	2.02	0.87
Q05193	Dynamin-1	0.758	1.37	1.65	1.20
P06748	Nucleophosmin	0.777	0.69	0.83	1.21
P80723	Brain acid soluble protein 1	0.787	1.79	1.62	0.90
P35527	Keratin, type I cytoskeletal 9	0.819	0.51	0.62	1.20
P31146	Coronin-1A	0.842	1.56	1.37	0.87
Q16555	Dihydropyrimidinase-related protein 2	0.851	1.55	1.40	0.90
P32119	Peroxiredoxin-2	0.874	1.35	1.23	0.91
P61764	Syntaxin-binding protein 1	0.875	1.81	1.94	1.07
Q8N111	Cell cycle exit and neuronal differentiation protein 1	0.900	1.25	1.16	0.92
P12532	Creatine kinase U-type, mitochondrial	0.955	1.37	1.38	1.01
Q08209	Serine threonine phosphatase 2B cat subunit-β isoform	0.962	1.39	1.41	1.02
P04264	Keratin, type II cytoskeletal 1	0.972	0.33	0.32	0.98

*Proteins were quantified in ≥2 cases from each group (Alzheimer’s disease and control). The table is sorted on *p*-value from group comparisons (red). The ratio between the AD and control group mean is shown in the right column (blue). Creatine kinase B-type, heat shock cognate 71, and 14-3-3 protein gamma (bolded) were studied further with IHC. AD, Alzheimer’s disease; IHC, immunohistochemistry; LCM, laser capture microdissection.*

### Immunohistochemistry

Thin sections of paraffinized brain tissue samples (8 μm) from the hippocampus and frontal cortex of patients with AD and control cases were deparaffinized and hydrated, first in xylene (Histolab, Spånga, Sweden) and then in a decreasing concentration of ethanol (99.5, 95, and 70% w/v) before rinsing in dH_2_O. Samples were autoclaved for antigen retrieval in DIVA decloaker bath (Thermo Fisher Scientific, Waltham, MA, United States) in a pressure cooker at +110°C for 20 min before being rinsed with dH_2_O. The IHC protocol was based on the MACH1 universal HRP-polymer kit (1-800-799-9499, BioCare Medical, Pacheco, CA, United States). In brief, the samples were first incubated with Peroxidazed1 (BioCare Medical) blocking agent for 5 min at RT, washed 3 × 5 min with phosphate-buffered saline with 0.05% Tween (PBS-T; Sigma-Aldrich), incubated with Background Sniper blocking reagent (Histolab) for 15 min at RT, washed 3 × 5 min with PBS-T. Following wash, the samples were incubated with primary antibodies from Novus Biological (Minneapolis, MN, United States); anti-creatine kinase B (#1-84 460, 1:200), anti-HSPA8/Hsc71 (#NBP 1-97 868, 1:400), and anti-14-3-3-γ (#NB100-407, 1:100) diluted in PBS at +4°C over-night. After washing for 3 × 5 min with PBS-T, incubation with secondary antibodies was performed with HRP-labeled polymer for 2 h at RT. After secondary polymer incubation, samples were washed with PBS-T 3 × 5 min, before being incubated with a 3,3′-Diaminobenzidine-Chromogen (DAB) solution for 5 min at RT. The samples were rinsed with dH_2_O before being dehydrated in a rising concentration of ethanol and placed in a xylene bath before mounting. Samples were mounted using a xylene-based mounting medium (Vectamount, Thermo Fisher Scientific), covered with coverslip panels with a refractive index of 1 and observed using light microscopy within 48 h of mounting. Samples were stored dry and dark when not in use. All results were duplicated to validate findings. Negative controls were performed for all antibodies by omitting the primary antibody (Supplementary Figure 1).

### Immunohistochemistry Image Acquisition

Immunohistochemistry pictures of DAB-chromogen-stained brain sections were captured using a Nikon Camera DS-Qi2 on a Nikon Eclipse E800 brightfield microscope (Nikon, Tokyo, Japan) with capture software Nikon NIS-elements. Color correction channels were adjusted for whitening by adjusting RGB channels, and conditions were kept the same for each image capture. The image capture center was localized at the hippocampal and cortex subregions of interest and images were captured using 10×, 20×, and 40× objectives.

### Quantification and Statistical Analysis of Immunohistochemistry

Quantification of signal intensity in IHC images was done using ImageJ imaging software (NIH, Bethesda, MD, United States). To derive quantification from IHC images, each pixel is given a value between 0 and 250 with 250 being the highest detectable intensity. Background signal was determined and removed by the measuring of negative control samples and removal of the corresponding intensity interval in all analyses. The CA1 and CA3 anatomical subareas of the hippocampus were examined separately. The neuronal signal was measured by drawing a mask around each individual neuron in each subarea. An average of 60 neurons was included from each patient per subarea. The mask area was normalized for size across all neurons, and mean signal intensity was then recorded. The neuronal signal was then excluded from subarea analysis to identify the non-neuronal signal. A positive signal was detected based on mean signal intensity in each sample area. The regional signal was defined as the whole signal detectable in the anatomically identified area. Neurons were identified according to the presence of cytoplasm around the nucleus, nuclear shape, and proximal apical dendrites. Neuronal degenerative status was determined using cytosolic volume, presence of dystrophic neurites, and atrophic apical dendrites. Glial cells were detected based on the absence of cytoplasm, the presence of cytoplasmic granular inclusions, and cell volume. The neuronal signal is displayed as the mean signal of 60 neurons per subarea. The signal intensity of samples is displayed in an interval of 0–250. Neuronal signal and subarea signal for each protein were compared between AD and controls. For quantification of the IHC, staining data of signal intensity were obtained with ImageJ, and statistical analysis was performed using Graphpad Prism software (Graphpad Software, San Diego, CA, United States). Unpaired Student’s *t*-tests were used to determine significance between AD and controls. A *p*-value <0.05 was deemed significant.

## Results

### The Cornu Ammonis 1 Pyramidal Cell Proteome Differs Between Alzheimer’s Disease and Controls

**FIGURE 2:**
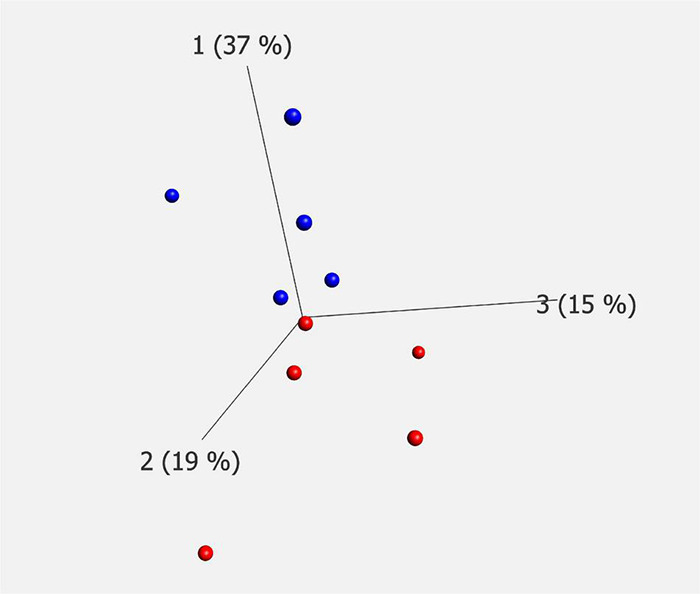
PCA plot of mass spectrometry data. Proteomic analysis of LCM material showed significant differences in protein constitution between AD (red) and controls (blue). Endpoint shows the percentage of variance. The analysis was performed on proteins containing two or more corresponding data points for each of AD and controls, *p* = 0.0023. PCA, principal component analysis; LCM, laser capture microdissection; AD, Alzheimer’s disease.

The demographics of the cases analyzed by each technique are summarized in Table 1. Donated brain specimens from AD and control cases were age and gender matched, and PMI was equally distributed among the cases. In the LCM analyzed cases, only one AD had known ApoE3/E4 status, but in the IHC analyses, a majority of the AD cases were E3/E4 carriers whereas none of the control cases had this genotype. Although the included control cases were neurologically healthy at the time of death, a majority of the cases had some level of AD-like pathology (Braak I/II and amyloid stage A). From each case, 1,000 individual neurons of the CA1 region of the hippocampus were isolated by LCM (Figure 1) and collected for protein analysis. After mixing with an internal ^18^O-labeled standard, the proteome was analyzed for each case using LC-MS/MS. Despite a low amount of starting material, a total of 470 unique proteins were identified in the analysis, still a majority was identified in less than two cases from each group (AD and control; Supplementary Table 1). Sixty-two proteins were identified in at least two cases from each group (Table 2). Of these, 30 proteins were found upregulated in AD and the mean ratio for all proteins in Table 2 is 1.3 indicating that for the approximate half of the proteins upregulated in AD, the mean difference between AD and control was larger than in those downregulated in AD. The individual protein levels from Table 2 were further included in a PCA to determine dataset differences in the overall proteomes between groups. Endpoint percent displays percentage of variance. There was a clear clustering in the general proteome of AD (red symbols) vs. control (blue), *p* = 0.0023 (Figure 2).

The top 10 proteins with the most differences in level between AD and control (excluding keratin which is a contaminant) included the previously well-studied inflammatory marker, glial fibrillary protein (GFAP) (Laurent et al., 2018), and several markers of neurodegeneration; neurofilament light polypeptide (NfL), microtubule-associated protein-2 (MAP2), spectrin β-chain and β-chain, and β-synuclein (Yan et al., 2012; Bacioglu et al., 2016; Halbgebauer et al., 2020). Although not largely studied in AD, malate dehydrogenase expression has been explored in different brain regions of AD previously (Zahid et al., 2014), thus we selected CKB, Hsc71, and 14-3-3-γ for downstream analyses since, to our knowledge, these proteins have been less studied in AD human brain tissue.

### Immunohistochemistry Analysis Confirmed the Laser Capture Microdissection-Proteomic Data on Creatine Kinase B-Type, 14-3-3-γ, and Hsc71

Immunohistochemistry was first performed on hippocampal brain tissue sections of AD and control cases to validate and further explore the proteomic results. The analysis was performed using samples from other donors than those used for the LCM analysis to investigate if our findings were valid also for other AD cases than those examined by proteomics. For this purpose, hippocampal sections were stained for CKB, 14-3-3-γ and Hsc71. Furthermore, we investigated whether the altered expression could be observed also in another AD-affected area and included the frontal cortex in our analysis. Differences in distribution patterns between regions and also subcellular and extracellular distribution are described below.

### Creatine Kinase B-Type Level Is Increased in Pyramidal Cells of Both Hippocampus and Frontal Cortex

**FIGURE 3:**
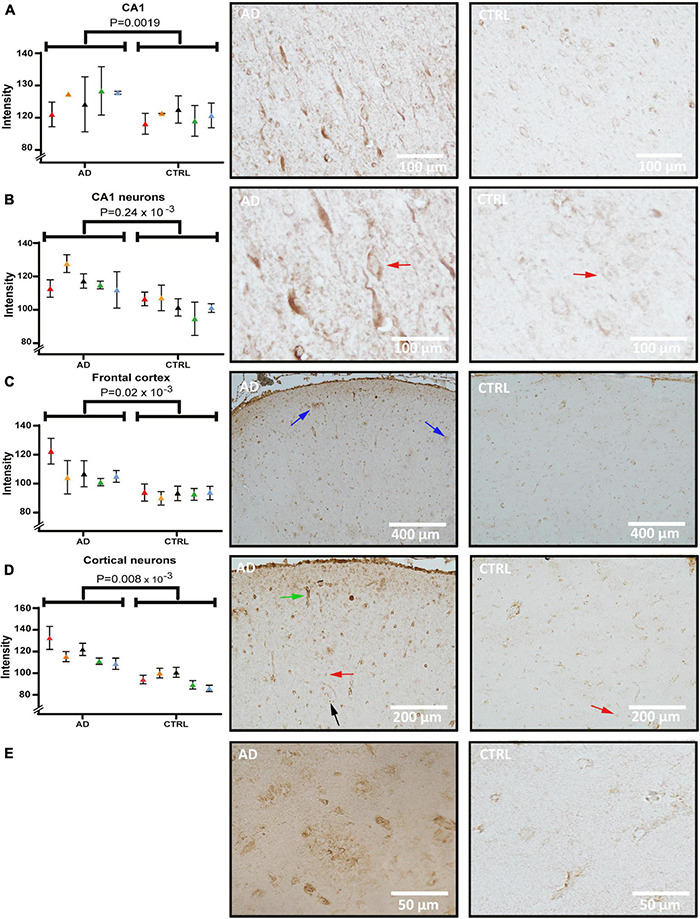
Creatine kinase B (CKB) levels and distribution in the hippocampus and frontal cortex of AD and control. Panel **(A)** shows quantification and brightfield images of CKB in the CA1 subfield of AD and control. Panel **(B)** shows quantification and analysis of CA1 pyramidal cellular soma of AD and control. Panel **(C)** shows quantification and images from the frontal cortex and panel **(D)** quantification and images of pyramidal cellular staining in the frontal cortex. Individual patients are indicated by colors in the graphs. Examples of pyramidal cells and glial cells are indicated by red and black arrows, respectively. CKB deposits within the frontal cortex were seen occasionally and is indicated by blue arrows in panel C and in magnified pictures from AD and control in panel **(E)**. Example of vascular staining that was seen in AD is indicated by a green arrow. AD, Alzheimer’s disease.

Figure 3 shows CKB staining in sections of the hippocampus and frontal cortex from control and AD cases. The CKB staining in neurons was predominantly somatic and evenly distributed in the proximal part of the apical dendrite and basal dendrites. Diffuse deposit-like staining was seen throughout the hippocampal subfield. The signal was quantified and found to be significantly elevated in the CA1 subfield of AD (Figure 3A, *p* = 1.9 × 10^–3^). When measuring the CKB signal in CA1 pyramidal cells (red arrows), an increased signal was seen in AD (Figure 3B, *p* = 0.24 × 10^–3^). In the frontal cortex, CKB was elevated in AD both in the whole of the investigated frontal cortical area (Figure 3C, *p* = 0.02 × 10^–3^) and within the cortical, pyramidal cell soma (Figure 3D, *p* = 0.008 × 10^–3^) compared to controls. Interestingly, CKB levels in the frontal cortex of patients with AD exhibited intense staining in deposit-like structures like those seen in the hippocampal structures. These deposits were not layer-specific and occurred throughout the frontal cortex (Figure 3C, indicated by blue arrows). Their appearance may be reminiscent of neuritic plaque formations (see high-magnification micrographs in panel E). Furthermore, capillaries (Figure 3D, green arrow) were intensively stained in AD tissue. Glial cells were also found positive (black arrow) and the white matter showed intense staining (data not shown).

### The 14-3-3-γ Level Is Increased in Pyramidal Cells of the Hippocampus and Frontal Cortex

**FIGURE 4:**
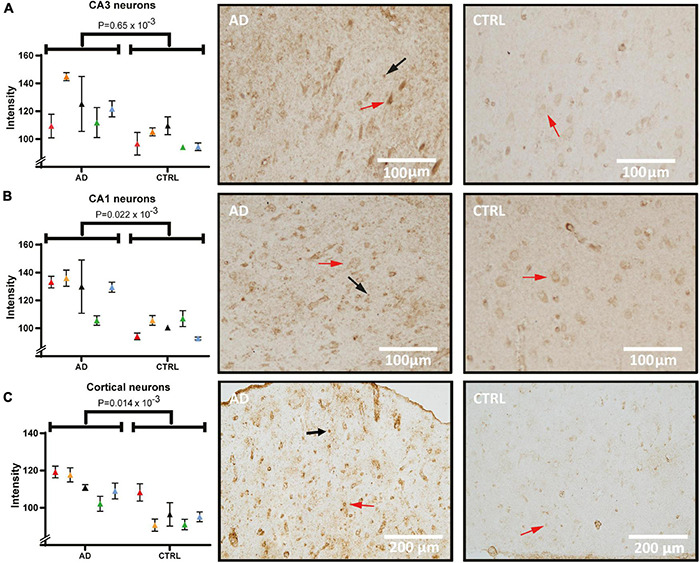
14-3-3-γ levels and distribution in pyramidal cells of the hippocampus and frontal cortex of AD and control. Panel **(A)** shows staining intensity of 14-3-3-γ in CA3 neurons quantified and compared between AD and control group, and tissue examples from one AD and one control case. Panel **(B)** shows staining intensity in quantified CA1 neurons and examples of analyzed images. Panel **(C)** shows frontal cortex quantification and staining. Individual patients are indicated by colors in the graphs. Examples of pyramidal cells and glia cells are indicated by red and black arrows respectively. AD, Alzheimer’s disease; CA1, cornu ammonis 1.

Figure 4 shows the immunostaining of 14-3-3-γ in sections from the hippocampus and frontal cortex. Semi-quantitative measures from the signal acquired from pyramidal cells (red arrows) show a significant difference between AD and controls in CA3 (Figure 4A, *p* = 0.65 × 10^–3^). In CA1 neurons, the staining pattern was similar to CA3, showing an increased intensity in AD (Figure 4B, *p* = 0.02 × 10^–3^). Example micrographs of CA3 and CA1 from each diagnostic group (right panel) illustrate that the staining was localized predominantly in the soma and with additional occasional nuclear staining in the pyramidal cells. Intense staining of glial cells was seen in AD in particular (black arrows) indicating an accumulation within the neuronal supporting cells. Considering the accumulated neuronal signal with a degenerative morphology in the hippocampus, there was a large variance between AD cases. Having detected strong pyramidal staining in the hippocampus, we decided to examine another heavily affected region in the AD brain that is rich in pyramidal cells; the frontal cortex. In concordance with the hippocampus, pyramidal cells of the AD frontal cortex show a significant, overall increase in 14-3-3-γ compared to the control group (Figure 4C, *p* = 0.014 × 10^–3^).

### Pyramidal Cells of the Alzheimer’s Disease Hippocampus Show Increased Levels of Hsc71

**FIGURE 5:**
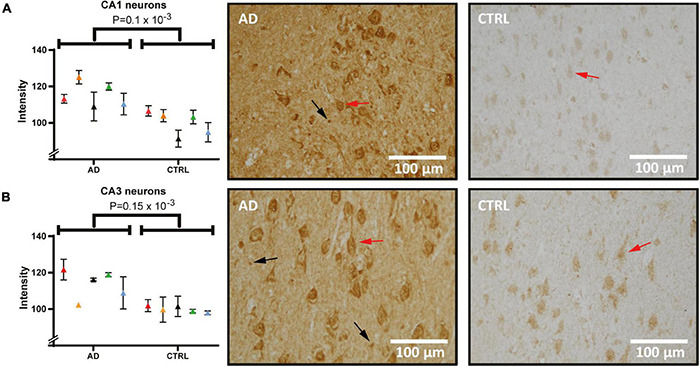
Heat shock cognate 71 (Hsc71) levels and distribution in the hippocampus of AD and control. Panel **(A)** shows staining of Hsc71 in CA1 neurons quantified and compared between AD and controls (individual patients are indicated by colors in the graph) and tissue examples from one AD and one control case. Panel **(B)** shows staining intensity in quantified CA3 neurons and examples of analyzed images. Examples of pyramidal cells and glia cells are indicated by red and black arrows respectively. AD, Alzheimer’s disease; CA1, cornu ammonis 1.

Next, hippocampal sections were stained and quantified for Hsc71 (Figure 5). In micrographs from both CA1 (Figure 5A) and CA3 (Figure 5B), the staining was mostly located in the cytoplasmic compartment of the neurons with increased intensity next to the plasma membrane. Furthermore, there were differences in staining intensity with more signal in neurons that have clear signs of degeneration. In one AD case, there was no detectable difference in the CA3 signal to the control signal. Yet, when analyzing pyramidal cell staining (red arrows) and comparing between groups, the Hsc71 signal was increased in AD in both hippocampal CA1 and CA3 (Figure 5A, *p* = 0.1 × 10^–3^ and Figure 5B, *p* = 0.15 × 10^–3^). In CA1, Hsc71 intensity was stronger in proximal apical dendrites when compared to CA3 (Figure 5A vs. Figure 5B). Glia cells can be noted to have accumulated the Hsc71 signal in both CA1 and CA3 (arrows). No difference in Hsc71 signal in the frontal cortex was found between AD and controls. In fact, Hsc71 did not localize specifically to any visible cells by IHC in this region (data not shown).

## Discussion

In the current approach of LCM-isolated neuronal soma proteomics, we reduce the complexity of the analyses by focusing on one cell type—the pyramidal CA1 cells. In the overall comparison between AD and control, the PCA analysis revealed a significant proteomic difference between groups. This highlights the fact that multiple proteins and signaling pathways of pyramidal AD CA1 cells are affected, at least during the final stages of the disease.

The most significant difference in protein level between AD and control in this study was seen for CKB. By IHC, CKB levels of CA1 pyramidal cells were confirmed in the hippocampus and further explored in the frontal cortex, where an increase in AD and intensely stained deposits were seen. CKB has previously been found to accumulate in the insoluble fraction from the AD brain (Kepchia et al., 2020), still knowledge about its normal cellular expression pattern varies between studies (Friedman and Roberts, 1994; Kaldis et al., 1996; Jost et al., 2002). One theory to explain the source of CKB deposits seen in our study could be leakage from dead cells. Indeed, CKB was present particularly in pyramidal cells with a late degenerative stage morphology, and increased levels were seen both within cells and in the extracellular space of AD. Another possibility would be extracellular secretion of CKB from oligodendrocytes, which produce and utilize large amounts of creatine (Manos et al., 1991; Tachikawa et al., 2004; Zhang et al., 2014).

Also among the detected proteins of our LCM, proteomic analysis was 14-3-3-γ which was seen to be specifically altered in AD pyramidal cells within the hippocampus but also in the frontal cortex. No extracellular deposits were seen in any of the studied cases, thus separating γ from some of the other isoforms found to accumulate throughout the brain in pathological conditions (Umahara et al., 2004; Shimada et al., 2013). Some variance in protein levels of 14-3-3-γ between the AD cases was seen, which could be related to previous findings from case studies of rapid onset dementias where higher 14-3-3 levels were associated with fast progression (Huang et al., 2003; Reinwald et al., 2004; Jayaratnam et al., 2008). Since 14-3-3 and its isoforms, such as 14-3-3-γ are associated with neuronal damage it is reasonable to suspect that 14-3-3-γ levels in the AD brain might reflect the speed of disease progression, although another study design than the current is needed to investigate this further.

CA 1 receives input from the temporoammonic path and the tri-synaptic pathway via Schaffer collaterals (Aksoy-Aksel and Manahan-Vaughan, 2013). The tri-synaptic pathway has been suggested as a specific processor of synaptic plasticity in CA1 and is increased during times of metabolic stress (Moeller et al., 1996). Furthermore, the frontal cortex is affected by metabolic changes and is known to contain a remarkable capability for synaptic plasticity (McEwen and Morrison, 2013). Interestingly, CKB is highly associated with cellular metabolism (Saks et al., 1978; Schlattner et al., 2016). Our results thus indicate that increased neuronal production of CKB takes place in areas with significant synaptic plasticity and is perhaps linked to a more general AD process, such as oxidation or metabolic stress. Hence, lowered glucose metabolism as commonly seen in AD (Kuehn, 2020) could give an explanation to increased CKB levels in the AD brain. More studies regarding the role of CKB activity in AD are warranted to determine whether the CKB increase is detrimental or indeed a compensatory/protective mechanism. As protective response, creatine supplementation is both affordable and safe to consider as a potential treatment strategy (Smith et al., 2014; AliMohammadi et al., 2015).

Although various chaperones, such as Hsc71, are known to be present in the brain (Koren et al., 2009), neither any study on chaperone distribution in individual neurons nor the specific distribution of Hsc71 in AD brain has, to our knowledge, previously been investigated. Hsc71 is normally found in the nucleus and at the plasma membrane level. In the current study, we show that the Hsc71 signal was evidently increased in AD, located throughout CA1 and CA3 pyramidal cells and with a deviating distribution pattern from its normal subcellular location. Although the nucleus was strongly stained in most cells, cytoplasmic staining was also present.

The Hsc71 signal was specific to the hippocampus as we did not find any Hsc71 protein staining in the frontal cortex. Further, neither extracellular accumulation of Hsc71 was seen within the cortex nor the hippocampus. There was also some neuropil positivity which appeared to stem from glial cells. Since the Hsp70 protein family is stress-activated neuroprotectors, it is reasonable to assume that upregulated Hsc71 is a compensatory mechanism to cellular stress in AD neurons (Kim et al., 2020). Other members of the Hsp70 family have been shown to accumulate extracellularly, but Hsc71 seems to be an exception since this chaperone was specifically accumulated intracellularly in AD hippocampal neurons. This prompts further investigation into Hsc71 and the implications of its possible neuroprotective abilities.

The current study has a few limitations; compared to proteomic studies of homogenates or micro-dissected larger hippocampal areas, the dissection of single pyramidal cells is a time-consuming process and in the end, relatively few proteins could be identified using this approach. Downstream analyses must be highly sensitive, and the findings should ultimately be explored further using other methods. Further, since mainly proteins present in the soma can be dissected, any changes in axons or neurites are not taken into account in this analysis, still the IHC analyses provide the possibility to explore more of the entire neuron. The strength, which makes the LCM approach quite unique, is that the analysis is specific to pyramidal cells and no other cell types, which should increase the impact of the findings. In line with this notion is our previous study using LCM, in which we showed differences in protein expression that could not be revealed by bulk analysis (Hashimoto et al., 2012b). A final limitation is that the majority of the control cases of this study were not completely free from pathology, still with the current study design we could not investigate if there were associations between Tau or amyloid load with CKB, 14-3-3-γ or Hsc71 intensity.

Since the micro-dissected AD cells are at the latest stages of the disease and thus the cells that have been most persistent to the disease, it is reasonable to believe that both protective and detrimental cellular signaling machineries are activated. CKB and Hsc71 are likely protective, or compensatory proteins, based on their respective functions. These could thus serve as potential new treatment candidates and should be explored in earlier disease stages, since neuronal protective mechanisms maybe diverse throughout disease progression. 14-3-3-γ on the other hand may represent a neuronal damage marker. Still, how the γ isoform differs from its protein family remains to be explored.

In conclusion, we found significant changes in the pyramidal cell proteome between AD and controls. By subsequent IHC of three of the most altered proteins between groups and with relatively few prior studies in the AD brain, we confirmed the proteomic findings. The most significant changes between AD and control in the proteomic analysis were seen for CKB, and particularly in pyramidal cells with a degenerative phenotype. We hypothesize that these changes could be due to metabolic changes. Our analysis further demonstrated that 14-3-3-γ, with previous conflicting data on levels in AD, was herein increased in AD pyramidal cells. Moreover, in contrast to the other 14-3-3 epitopes, 14-3-3-γ did not form extracellular deposits in our analysis. The chaperone Hsc71 was shown to be increased in AD brain and its localization was spread from the normal, nuclear compartment to the cytoplasm in hippocampal cells. Based on the roles of CKB and Hsc71 in cellular protection, it is possible that the most pathology-resistant neurons of the hippocampus have a particular ability to upregulate these proteins. Thus, it maybe relevant to investigate these putative AD-protective responses as future drug targets.

## Data Availability Statement

All data from this study are in Table 2 and Supplementary Tables 1, 2. The mass spectrometry proteomics data have been deposited to the ProteomeXchange Consortium via the PRIDE partner repository with the dataset identifier PXD028282 (Perez-Riverol et al., 2019).

## Ethics Statement

This study involving post-mortem human brain was conducted under the auspice and in accordance with the ethical standards of the institutional and national regional research committee “Regional Ethics Review Board in Stockholm” (ethical permit nr 2013/1301-31/2) and with the 1964 Helsinki declaration and its later amendments or comparable ethical standards. All material has been collected from donors whose written informed consent for brain autopsy and the use of material and clinical information for research purposes has been obtained by the Netherlands brain bank (NBB). All AD subjects met the criteria for definitive AD according to the Consortium to Establish a Registry for AD (Morris et al., 1989). The control subjects had no known psychiatric or neurological disorder.

## Author Contributions

AS-M, BW, NB, and LT designed the study. AS-M conducted the LCM and sample preparations for proteomics. PN and LT analyzed the proteomic data. MA performed the IHC experiments, acquired the images, and analyzed the data with support from SS-W. AS-M and MA wrote the first draft of the manuscript and prepared the figures. All authors read and approved the final manuscript.

## Conflict of Interest

The authors declare that the research was conducted in the absence of any commercial or financial relationships that could be construed as a potential conflict of interest.

## Publisher’s Note

All claims expressed in this article are solely those of the authors and do not necessarily represent those of their affiliated organizations, or those of the publisher, the editors and the reviewers. Any product that may be evaluated in this article, or claim that may be made by its manufacturer, is not guaranteed or endorsed by the publisher.

## Supplementary Material

Supplementary Figure 1 Negative controls from the respective experiments when analyzing CKB, 14-3-3-γ and Hsc71 by immunohistochemistry. CKB, Creatine kinase B; Hsc71, heat shock cognate.

Supplementary Table 1 In total, 470 proteins were identified from the proteomic analysis of the LCM extracted CA1 neurons (10 cases). Most of the proteins were identified in less than two cases from each group (AD and control). Proteins are sorted based on the difference between AD and controls. LCM, laser capture microdissection**;** CA1, cornu ammonis 1; AD: Alzheimer’s disease.

Supplementary Table 2 Individual lists of the proteins identified from the mass spectrometric analysis including the number of peptides identified for each case (1–5). C: control; AD: Alzheimer’s disease.

## Abbreviations

A β: Amyloid β -peptide
AD: Alzheimer’s disease
APP: amyloid precursor protein
CKB: Creatine Kinase B-type
CA1: Cornu ammonis 1
CA3: Cornu ammonis 3
CJD: Creutzfeldt Jakob’s disease
DAB: 3,3′-Diaminobenzidine
IHC: Immunohistochemistry
Hsp70: 70 kilo Dalton heat shock protein
Hsc71: Heat shock cognate protein 71
LCM: Laser capture microdissection
NBB: Netherlands brain bank
NFT: Neurofibrillary tangles
PBS: Phosphate buffered saline
PCA: Principal component analysis
PMI: Post mortem interval.
