# Supplementary figures and images for: Microdissected Pyramidal Cell Proteomics of Alzheimer Brain Reveals Alterations in Creatine Kinase B-Type, 14-3-3-γ, and Heat Shock Cognate 71
Source: Front Aging Neurosci. 2021 Nov 19;13:735334. doi: 10.3389/fnagi.2021.735334 (PMC8641652; doi:10.3389/fnagi.2021.735334)

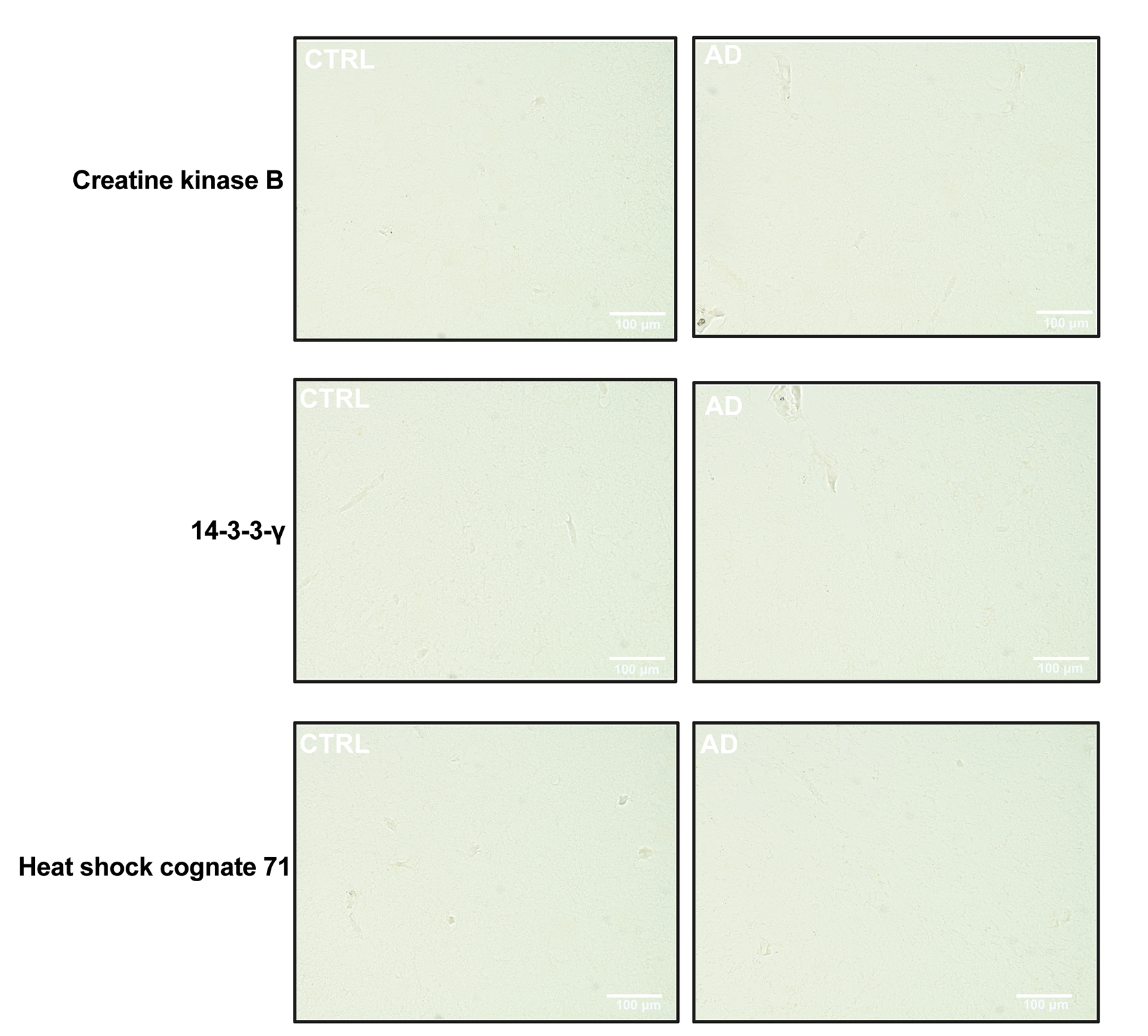

Supplement: Supplementary Figure 1 — Negative controls from the respective experiments when analyzing CKB, 14-3-3-γ and Hsc71 by immunohistochemistry. CKB, Creatine kinase B; Hsc71, heat shock cognate. [file Image_1.png]
