# Supplementary material for: Microdissected Pyramidal Cell Proteomics of Alzheimer Brain Reveals Alterations in Creatine Kinase B-Type, 14-3-3-γ, and Heat Shock Cognate 71
Source: Front Aging Neurosci. 2021 Nov 19;13:735334. doi: 10.3389/fnagi.2021.735334 (PMC8641652; doi:10.3389/fnagi.2021.735334)
Supplement: Supplementary Table 1 — In total, 470 proteins were identified from the proteomic analysis of the LCM extracted CA1 neurons (10 cases). Most of the proteins were identified in less than two cases from each group (AD and control). Proteins are sorted based on the difference between AD and controls. LCM, laser capture microdissection; CA1, cornu ammonis 1; AD: Alzheimer’s disease. [file Table_1.DOCX]

**Table S1: All proteins detected by proteomics in LCM fractions**

|  |  |  | Average STD/ | Average STD/ | Ratio |
| --- | --- | --- | --- | --- | --- |
| **ID** | **Protein** | **P** | **AD** | **Cntrl** | **AD/Cntrl** |
| P12277 | Creatine kinase B-type | 0,004 | 1,38 | 2,30 | 1,67 |
| Q13813 | Spectrin alpha chain, non-erythrocytic 1 | 0,010 | 1,05 | 1,72 | 1,63 |
| P14136 | Glial fibrillary acidic protein | 0,051 | 2,17 | 3,56 | 1,64 |
| P07196 | Neurofilament light polypeptide | 0,104 | 0,53 | 1,33 | 2,52 |
| Q01082 | Spectrin beta chain, non-erythrocytic 1 | 0,116 | 1,58 | 0,86 | 0,54 |
| Q16143 | Beta-synuclein | 0,136 | 1,72 | 0,53 | 0,31 |
| P11137 | Microtubule-associated protein 2 | 0,171 | 1,06 | 0,38 | 0,35 |
| P40925 | Malate dehydrogenase, cytoplasmic | 0,181 | 0,55 | 1,60 | 2,91 |
| P35908 | Keratin, type II cytoskeletal 2 epidermal | 0,189 | 0,22 | 1,50 | 6,93 |
| P11142 | Heat shock cognate 71 kDa protein | 0,192 | 0,72 | 1,53 | 2,11 |
| P13645 | Keratin, type I cytoskeletal 10 | 0,236 | 0,53 | 1,07 | 2,02 |
| P61981 | 14-3-3 protein gamma | 0,240 | 0,62 | 1,86 | 3,01 |
| P09936 | Ubiquitin carboxyl-terminal hydrolase isozyme L1 | 0,254 | 2,26 | 1,73 | 0,77 |
| Q16720 | Plasma membrane calcium-transporting ATPase 3 | 0,264 | 2,48 | 1,36 | 0,55 |
| P00338 | L-lactate dehydrogenase A chain | 0,274 | 0,29 | 0,75 | 2,60 |
| Q9UQM7 | Calcium/calmodulin-dependent protein kinase type II subunit alpha | 0,295 | 1,54 | 1,11 | 0,72 |
| P07900 | Heat shock protein HSP 90-alpha | 0,305 | 1,63 | 1,09 | 0,67 |
| P22626 | Heterogeneous nuclear ribonucleoproteins A2/B1 | 0,328 | 0,61 | 1,24 | 2,04 |
| P19367 | Hexokinase-1 | 0,332 | 1,19 | 0,97 | 0,82 |
| Q13423 | NAD(P) transhydrogenase, mitochondrial | 0,355 | 1,64 | 0,78 | 0,48 |
| O43301 | Heat shock 70 kDa protein 12A | 0,358 | 0,53 | 2,62 | 4,97 |
| P06576 | ATP synthase subunit beta, mitochondrial | 0,364 | 3,51 | 1,89 | 0,54 |
| P17600 | Synapsin-1 | 0,371 | 0,93 | 0,72 | 0,77 |
| P05129 | Protein kinase C gamma type | 0,373 | 0,69 | 0,79 | 1,15 |
| P68366 | Tubulin alpha-4A chain | 0,379 | 0,86 | 1,27 | 1,47 |
| P10809 | 60 kDa heat shock protein, mitochondrial | 0,389 | 5,47 | 1,41 | 0,26 |
| P38606 | V-type proton ATPase catalytic subunit A | 0,407 | 0,82 | 1,14 | 1,38 |
| P00505 | Aspartate aminotransferase, mitochondrial | 0,418 | 0,77 | 1,90 | 2,47 |
| P04350 | Tubulin beta-4A chain | 0,423 | 1,54 | 2,11 | 1,37 |
| P68104 | Elongation factor 1-alpha 1 | 0,450 | 0,55 | 1,08 | 1,96 |
| Q13885 | Tubulin beta-2A chain | 0,464 | 2,00 | 1,25 | 0,62 |
| P10636 | Microtubule-associated protein tau | 0,469 | 1,26 | 0,98 | 0,78 |
| P63104 | 14-3-3 protein zeta/delta | 0,471 | 1,14 | 1,50 | 1,31 |
| P46459 | Vesicle-fusing ATPase | 0,476 | 1,46 | 1,26 | 0,87 |
| P27348 | 14-3-3 protein theta | 0,488 | 1,84 | 1,24 | 0,68 |
| P69905 | Hemoglobin subunit alpha | 0,495 | 1,21 | 0,99 | 0,81 |
| P06744 | Glucose-6-phosphate isomerase | 0,496 | 1,47 | 1,14 | 0,77 |
| P62158 | Calmodulin | 0,530 | 4,27 | 1,78 | 0,42 |
| P13637 | Sodium/potassium-transporting ATPase subunit alpha-3 | 0,553 | 3,08 | 1,91 | 0,62 |
| P02686 | Myelin basic protein | 0,553 | 2,25 | 1,76 | 0,78 |
| P07195 | L-lactate dehydrogenase B chain | 0,575 | 1,29 | 2,31 | 1,80 |
| P25705 | ATP synthase subunit alpha, mitochondrial | 0,618 | 0,89 | 1,03 | 1,15 |
| P60880 | Synaptosomal-associated protein 25 | 0,641 | 1,36 | 1,54 | 1,13 |
| P60201 | Myelin proteolipid protein | 0,666 | 1,78 | 1,54 | 0,86 |
| Q6NXT2 | Histone H3.3C | 0,667 | 0,61 | 0,68 | 1,11 |
| P23246 | Splicing factor, proline- and glutamine-rich | 0,709 | 0,49 | 0,66 | 1,34 |
| P07339 | Cathepsin D | 0,719 | 1,21 | 0,95 | 0,79 |
| P14618 | Pyruvate kinase isozymes M1/M2 | 0,726 | 1,54 | 1,41 | 0,92 |
| P21281 | V-type proton ATPase subunit B, brain isoform | 0,740 | 1,08 | 0,92 | 0,84 |
| P60709 | Actin, cytoplasmic 1 | 0,745 | 2,33 | 2,02 | 0,87 |
| Q05193 | Dynamin-1 | 0,758 | 1,37 | 1,65 | 1,20 |
| P06748 | Nucleophosmin | 0,777 | 0,69 | 0,83 | 1,21 |
| P80723 | Brain acid soluble protein 1 | 0,787 | 1,79 | 1,62 | 0,90 |
| P35527 | Keratin, type I cytoskeletal 9 | 0,819 | 0,51 | 0,62 | 1,20 |
| P31146 | Coronin-1A | 0,842 | 1,56 | 1,37 | 0,87 |
| Q16555 | Dihydropyrimidinase-related protein 2 | 0,851 | 1,55 | 1,40 | 0,90 |
| P32119 | Peroxiredoxin-2 | 0,874 | 1,35 | 1,23 | 0,91 |
| P61764 | Syntaxin-binding protein 1 | 0,875 | 1,81 | 1,94 | 1,07 |
| Q8N111 | Cell cycle exit and neuronal differentiation protein 1 | 0,900 | 1,25 | 1,16 | 0,92 |
| P12532 | Creatine kinase U-type, mitochondrial | 0,955 | 1,37 | 1,38 | 1,01 |
| Q08209 | Serine/threonine-protein phosphatase 2B catalytic subunit alpha isoform | 0,962 | 1,39 | 1,41 | 1,02 |
| P04264 | Keratin, type II cytoskeletal 1 | 0,972 | 0,33 | 0,32 | 0,98 |
| A4UGR9 | Xin actin-binding repeat-containing protein 2 | # | n/a | 0,31 | n/a |
| A6NHL2 | Tubulin alpha chain-like 3 | # | n/a | 1,22 | n/a |
| A8K2U0 | Alpha-2-macroglobulin-like protein 1 | # | n/a | 0,97 | n/a |
| O00154 | Cytosolic acyl coenzyme A thioester hydrolase | # | 2,75 | n/a | n/a |
| O00241 | Signal-regulatory protein beta-1 | # | 0,55 | n/a | n/a |
| O00264 | Membrane-associated progesterone receptor component 1 | # | 0,14 | n/a | n/a |
| O00401 | Neural Wiskott-Aldrich syndrome protein | # | n/a | 0,35 | n/a |
| O00422 | Histone deacetylase complex subunit SAP18 | # | n/a | 0,53 | n/a |
| O00429 | Dynamin-1-like protein | # | n/a | 0,88 | n/a |
| O00571 | ATP-dependent RNA helicase DDX3X | # | n/a | 0,70 | n/a |
| O14773 | Tripeptidyl-peptidase 1 | # | n/a | 0,97 | n/a |
| O15145 | Actin-related protein 2/3 complex subunit 3 | # | 0,97 | n/a | n/a |
| O15394 | Neural cell adhesion molecule 2 | # | 0,08 | n/a | n/a |
| O43175 | D-3-phosphoglycerate dehydrogenase | # | n/a | 1,03 | n/a |
| O43240 | Kallikrein-10 | # | n/a | 0,54 | n/a |
| O43312 | Metastasis suppressor protein 1 | # | n/a | 0,34 | n/a |
| O43390 | Heterogeneous nuclear ribonucleoprotein R | # | 3,73 | 1,18 | 0,32 |
| O43426 | Synaptojanin-1 | # | 0,36 | 1,39 | 3,81 |
| O43548 | Protein-glutamine gamma-glutamyltransferase 5 | # | n/a | 1,00 | n/a |
| O43759 | Synaptogyrin-1 | # | 2,69 | 1,50 | 0,56 |
| O43761 | Synaptogyrin-3 | # | n/a | 0,40 | n/a |
| O60234 | Glia maturation factor gamma | # | 1,05 | n/a | n/a |
| O60262 | Guanine nucleotide-binding protein G(I)/G(S)/G(O) subunit gamma-7 | # | n/a | 0,66 | n/a |
| O60502 | Bifunctional protein NCOAT | # | 0,71 | n/a | n/a |
| O60641 | Clathrin coat assembly protein AP180 | # | 4,51 | 1,60 | 0,35 |
| O60814 | Histone H2B type 1-K | # | n/a | 0,87 | n/a |
| O75037 | Kinesin-like protein KIF21B | # | n/a | 1,55 | n/a |
| O75083 | WD repeat-containing protein 1 | # | 0,28 | 0,69 | 2,46 |
| O75323 | Protein NipSnap homolog 2 | # | n/a | 0,78 | n/a |
| O75342 | Arachidonate 12-lipoxygenase, 12R-type | # | n/a | 0,85 | n/a |
| O75369 | Filamin-B | # | n/a | 1,12 | n/a |
| O75390 | Citrate synthase, mitochondrial | # | 1,83 | n/a | n/a |
| O75781 | Paralemmin-1 | # | 0,55 | 0,42 | 0,76 |
| O75964 | ATP synthase subunit g, mitochondrial | # | n/a | 1,94 | n/a |
| O76070 | Gamma-synuclein | # | n/a | 0,72 | n/a |
| O94760 | N(G),N(G)-dimethylarginine dimethylaminohydrolase 1 | # | 0,89 | 1,77 | 1,99 |
| O94772 | Lymphocyte antigen 6H | # | 0,88 | n/a | n/a |
| O94811 | Tubulin polymerization-promoting protein | # | 1,06 | n/a | n/a |
| O94925 | Glutaminase kidney isoform, mitochondrial | # | n/a | 0,16 | n/a |
| O95336 | 6-phosphogluconolactonase | # | n/a | 0,91 | n/a |
| O95373 | Importin-7 | # | n/a | 0,92 | n/a |
| O95674 | Phosphatidate cytidylyltransferase 2 | # | n/a | 1,54 | n/a |
| O95678 | Keratin, type II cytoskeletal 75 | # | 1,74 | n/a | n/a |
| O95741 | Copine-6 | # | 0,28 | n/a | n/a |
| P00367 | Glutamate dehydrogenase 1, mitochondrial | # | n/a | 1,63 | n/a |
| P00387 | NADH-cytochrome b5 reductase 3 | # | n/a | 1,60 | n/a |
| P00403 | Cytochrome c oxidase subunit 2 | # | 0,73 | 0,95 | 1,31 |
| P00441 | Superoxide dismutase [Cu-Zn] | # | n/a | 0,29 | n/a |
| P00558 | Phosphoglycerate kinase 1 | # | 0,81 | n/a | n/a |
| P00568 | Adenylate kinase isoenzyme 1 | # | n/a | 1,15 | n/a |
| P01620 | Ig kappa chain V-III region SIE | # | n/a | 1,52 | n/a |
| P01766 | Ig heavy chain V-III region BRO | # | n/a | 1,28 | n/a |
| P01833 | Polymeric immunoglobulin receptor | # | 0,19 | n/a | n/a |
| P01834 | Ig kappa chain C region | # | 0,47 | 1,95 | 4,11 |
| P01857 | Ig gamma-1 chain C region | # | 0,44 | 1,02 | 2,32 |
| P01859 | Ig gamma-2 chain C region | # | n/a | 7,44 | n/a |
| P01861 | Ig gamma-4 chain C region | # | n/a | 1,24 | n/a |
| P01876 | Ig alpha-1 chain C region | # | 1,32 | n/a | n/a |
| P02042 | Hemoglobin subunit delta | # | n/a | 0,89 | n/a |
| P02511 | Alpha-crystallin B chain | # | n/a | 0,09 | n/a |
| P02533 | Keratin, type I cytoskeletal 14 | # | 0,70 | 0,65 | 0,93 |
| P02538 | Keratin, type II cytoskeletal 6A | # | 0,50 | 0,72 | 1,44 |
| P02545 | Prelamin-A/C | # | 0,42 | 0,66 | 1,58 |
| P02675 | Fibrinogen beta chain | # | n/a | 0,25 | n/a |
| P02768 | Serum albumin | # | 0,34 | 0,63 | 1,85 |
| P02792 | Ferritin light chain | # | n/a | 1,07 | n/a |
| P04075 | Fructose-bisphosphate aldolase A | # | 2,18 | 1,57 | 0,72 |
| P04083 | Annexin A1 | # | n/a | 0,76 | n/a |
| P04179 | Superoxide dismutase [Mn], mitochondrial | # | 0,97 | n/a | n/a |
| P04259 | Keratin, type II cytoskeletal 6B | # | 0,43 | 1,46 | 3,36 |
| P04406 | Glyceraldehyde-3-phosphate dehydrogenase | # | 1,00 | 0,85 | 0,85 |
| P04792 | Heat shock protein beta-1 | # | 0,67 | 1,06 | 1,59 |
| P05023 | Sodium/potassium-transporting ATPase subunit alpha-1 | # | n/a | 0,94 | n/a |
| P05026 | Sodium/potassium-transporting ATPase subunit beta-1 | # | 3,76 | 1,48 | 0,39 |
| P05089 | Arginase-1 | # | n/a | 1,33 | n/a |
| P05091 | Aldehyde dehydrogenase, mitochondrial | # | n/a | 0,79 | n/a |
| P05141 | ADP/ATP translocase 2 | # | 2,16 | 0,87 | 0,40 |
| P05198 | Eukaryotic translation initiation factor 2 subunit 1 | # | 1,50 | n/a | n/a |
| P05413 | Fatty acid-binding protein, heart | # | 0,69 | 1,11 | 1,61 |
| P05496 | ATP synthase F(0) complex subunit C1, mitochondrial | # | 0,81 | 1,21 | 1,49 |
| P05787 | Keratin, type II cytoskeletal 8 | # | 2,42 | n/a | n/a |
| P06396 | Gelsolin | # | n/a | 0,64 | n/a |
| P06733 | Alpha-enolase | # | 0,14 | 1,05 | 7,69 |
| P06753 | Tropomyosin alpha-3 chain | # | n/a | 0,99 | n/a |
| P07197 | Neurofilament medium polypeptide | # | 1,12 | 0,67 | 0,59 |
| P07355 | Annexin A2 | # | n/a | 1,23 | n/a |
| P07437 | Tubulin beta chain | # | n/a | 6,46 | n/a |
| P07476 | Involucrin | # | n/a | 0,52 | n/a |
| P07737 | Profilin-1 | # | n/a | 1,38 | n/a |
| P07814 | Bifunctional glutamate/proline--tRNA ligase | # | n/a | 5,30 | n/a |
| P07910 | Heterogeneous nuclear ribonucleoproteins C1/C2 | # | n/a | 0,24 | n/a |
| P07954 | Fumarate hydratase, mitochondrial | # | n/a | 0,51 | n/a |
| P08107 | Heat shock 70 kDa protein 1A/1B | # | 0,52 | 0,76 | 1,47 |
| P08133 | Annexin A6 | # | n/a | 0,61 | n/a |
| P08237 | 6-phosphofructokinase, muscle type | # | 1,23 | n/a | n/a |
| P08238 | Heat shock protein HSP 90-beta | # | 0,62 | n/a | n/a |
| P08247 | Synaptophysin | # | 4,97 | n/a | n/a |
| P08559 | Pyruvate dehydrogenase E1 component subunit alpha, somatic form, mitochondrial | # | n/a | 2,68 | n/a |
| P08670 | Vimentin | # | 1,10 | n/a | n/a |
| P08758 | Annexin A5 | # | n/a | 1,39 | n/a |
| P08779 | Keratin, type I cytoskeletal 16 | # | 1,15 | 0,84 | 0,72 |
| P09104 | Gamma-enolase | # | 1,38 | 0,44 | 0,32 |
| P09211 | Glutathione S-transferase P | # | n/a | 0,73 | n/a |
| P09471 | Guanine nucleotide-binding protein G(o) subunit alpha | # | n/a | 2,27 | n/a |
| P09497 | Clathrin light chain B | # | n/a | 0,91 | n/a |
| P09525 | Annexin A4 | # | n/a | 0,77 | n/a |
| P09543 | 2',3'-cyclic-nucleotide 3'-phosphodiesterase | # | 1,58 | 4,05 | 2,56 |
| P09622 | Dihydrolipoyl dehydrogenase, mitochondrial | # | 0,73 | n/a | n/a |
| P09651 | Heterogeneous nuclear ribonucleoprotein A1 | # | n/a | 1,46 | n/a |
| P09972 | Fructose-bisphosphate aldolase C | # | 2,32 | 2,16 | 0,93 |
| P0C0S5 | Histone H2A.Z | # | 0,46 | 0,39 | 0,85 |
| P0CG05 | Ig lambda-2 chain C regions | # | 0,43 | n/a | n/a |
| P0CG48 | Polyubiquitin-C | # | 0,52 | 1,18 | 2,27 |
| P10114 | Ras-related protein Rap-2a | # | 1,03 | 0,32 | 0,31 |
| P10515 | Dihydrolipoyllysine-residue acetyltransferase component of pyruvate dehydrogenase complex, mitochondrial | # | n/a | 0,80 | n/a |
| P10599 | Thioredoxin | # | n/a | 0,22 | n/a |
| P10909 | Clusterin | # | n/a | 1,28 | n/a |
| P11021 | 78 kDa glucose-regulated protein | # | 0,46 | 1,32 | 2,89 |
| P11169 | Solute carrier family 2, facilitated glucose transporter member 3 | # | n/a | 0,73 | n/a |
| P11177 | Pyruvate dehydrogenase E1 component subunit beta, mitochondrial | # | 0,75 | 0,85 | 1,14 |
| P11216 | Glycogen phosphorylase, brain form | # | 0,72 | n/a | n/a |
| P11233 | Ras-related protein Ral-A | # | n/a | 2,06 | n/a |
| P12035 | Keratin, type II cytoskeletal 3 | # | n/a | 0,89 | n/a |
| P12036 | Neurofilament heavy polypeptide | # | 0,47 | n/a | n/a |
| P12235 | ADP/ATP translocase 1 | # | n/a | 1,64 | n/a |
| P12236 | ADP/ATP translocase 3 | # | n/a | 1,34 | n/a |
| P13489 | Ribonuclease inhibitor | # | n/a | 0,79 | n/a |
| P13591 | Neural cell adhesion molecule 1 | # | 0,54 | 0,83 | 1,53 |
| P13639 | Elongation factor 2 | # | n/a | 0,86 | n/a |
| P13647 | Keratin, type II cytoskeletal 5 | # | 0,35 | 0,56 | 1,60 |
| P13928 | Annexin A8 | # | n/a | 0,85 | n/a |
| P14406 | Cytochrome c oxidase subunit 7A2, mitochondrial | # | n/a | 2,98 | n/a |
| P14625 | Endoplasmin | # | 2,07 | 0,86 | 0,42 |
| P14735 | Insulin-degrading enzyme | # | n/a | 0,98 | n/a |
| P14866 | Heterogeneous nuclear ribonucleoprotein L | # | n/a | 0,51 | n/a |
| P14923 | Junction plakoglobin | # | n/a | 1,04 | n/a |
| P15259 | Phosphoglycerate mutase 2 | # | 1,37 | n/a | n/a |
| P15880 | 40S ribosomal protein S2 | # | n/a | 0,31 | n/a |
| P15924 | Desmoplakin | # | 0,47 | 0,66 | 1,42 |
| P15954 | Cytochrome c oxidase subunit 7C, mitochondrial | # | n/a | 0,80 | n/a |
| P16152 | Carbonyl reductase [NADPH] 1 | # | 2,80 | 0,89 | 0,32 |
| P16298 | Serine/threonine-protein phosphatase 2B catalytic subunit beta isoform | # | n/a | 0,35 | n/a |
| P16520 | Guanine nucleotide-binding protein G(I)/G(S)/G(T) subunit beta-3 | # | 0,59 | 1,57 | 2,67 |
| P16615 | Sarcoplasmic/endoplasmic reticulum calcium ATPase 2 | # | 1,06 | 1,52 | 1,43 |
| P17174 | Aspartate aminotransferase, cytoplasmic | # | 1,06 | 2,78 | 2,63 |
| P17252 | Protein kinase C alpha type | # | 1,40 | n/a | n/a |
| P17677 | Neuromodulin | # | 1,20 | 2,90 | 2,42 |
| P17858 | 6-phosphofructokinase, liver type | # | n/a | 2,92 | n/a |
| P17931 | Galectin-3 | # | n/a | 0,92 | n/a |
| P18085 | ADP-ribosylation factor 4 | # | 1,72 | 2,74 | 1,59 |
| P18124 | 60S ribosomal protein L7 | # | n/a | 0,42 | n/a |
| P19013 | Keratin, type II cytoskeletal 4 | # | n/a | 1,48 | n/a |
| P19338 | Nucleolin | # | 0,15 | 0,68 | 4,48 |
| P19404 | NADH dehydrogenase [ubiquinone] flavoprotein 2, mitochondrial | # | n/a | 1,71 | n/a |
| P20020 | Plasma membrane calcium-transporting ATPase 1 | # | n/a | 1,85 | n/a |
| P20073 | Annexin A7 | # | n/a | 0,80 | n/a |
| P20336 | Ras-related protein Rab-3A | # | 2,46 | 1,12 | 0,46 |
| P20674 | Cytochrome c oxidase subunit 5A, mitochondrial | # | n/a | 1,53 | n/a |
| P20930 | Filaggrin | # | n/a | 1,07 | n/a |
| P21333 | Filamin-A | # | n/a | 1,45 | n/a |
| P21579 | Synaptotagmin-1 | # | n/a | 2,50 | n/a |
| P21796 | Voltage-dependent anion-selective channel protein 1 | # | 1,58 | n/a | n/a |
| P22061 | Protein-L-isoaspartate(D-aspartate) O-methyltransferase | # | n/a | 1,02 | n/a |
| P22314 | Ubiquitin-like modifier-activating enzyme 1 | # | 0,44 | n/a | n/a |
| P22392 | Nucleoside diphosphate kinase B | # | n/a | 0,44 | n/a |
| P22695 | Cytochrome b-c1 complex subunit 2, mitochondrial | # | n/a | 0,77 | n/a |
| P22735 | Protein-glutamine gamma-glutamyltransferase K | # | n/a | 1,03 | n/a |
| P23368 | NAD-dependent malic enzyme, mitochondrial | # | 1,02 | n/a | n/a |
| P23471 | Receptor-type tyrosine-protein phosphatase zeta | # | n/a | 1,80 | n/a |
| P23526 | Adenosylhomocysteinase | # | n/a | 0,51 | n/a |
| P23588 | Eukaryotic translation initiation factor 4B | # | n/a | 1,02 | n/a |
| P24539 | ATP synthase subunit b, mitochondrial | # | 1,68 | 1,25 | 0,74 |
| P26641 | Elongation factor 1-gamma | # | 0,06 | 0,22 | 3,75 |
| P27797 | Calreticulin | # | 1,13 | n/a | n/a |
| P27816 | Microtubule-associated protein 4 | # | n/a | 0,49 | n/a |
| P27824 | Calnexin | # | n/a | 1,32 | n/a |
| P28066 | Proteasome subunit alpha type-5 | # | n/a | 0,71 | n/a |
| P28072 | Proteasome subunit beta type-6 | # | n/a | 0,56 | n/a |
| P28331 | NADH-ubiquinone oxidoreductase 75 kDa subunit, mitochondrial | # | n/a | 0,84 | n/a |
| P28482 | Mitogen-activated protein kinase 1 | # | 0,74 | 0,94 | 1,27 |
| P29401 | Transketolase | # | n/a | 1,14 | n/a |
| P29966 | Myristoylated alanine-rich C-kinase substrate | # | 1,29 | 1,05 | 0,81 |
| P30041 | Peroxiredoxin-6 | # | 0,51 | 1,55 | 3,06 |
| P30044 | Peroxiredoxin-5, mitochondrial | # | 3,93 | n/a | n/a |
| P30048 | Thioredoxin-dependent peroxide reductase, mitochondrial | # | n/a | 2,00 | n/a |
| P30049 | ATP synthase subunit delta, mitochondrial | # | 0,54 | 0,62 | 1,16 |
| P30084 | Enoyl-CoA hydratase, mitochondrial | # | 0,52 | 2,71 | 5,25 |
| P30086 | Phosphatidylethanolamine-binding protein 1 | # | 2,29 | n/a | n/a |
| P30101 | Protein disulfide-isomerase A3 | # | 0,84 | 0,46 | 0,55 |
| P30153 | Serine/threonine-protein phosphatase 2A 65 kDa regulatory subunit A alpha isoform | # | n/a | 1,57 | n/a |
| P31930 | Cytochrome b-c1 complex subunit 1, mitochondrial | # | n/a | 0,97 | n/a |
| P31942 | Heterogeneous nuclear ribonucleoprotein H3 | # | 0,33 | 0,54 | 1,64 |
| P31943 | Heterogeneous nuclear ribonucleoprotein H | # | n/a | 1,24 | n/a |
| P31944 | Caspase-14 | # | n/a | 0,63 | n/a |
| P31946 | 14-3-3 protein beta/alpha | # | 2,04 | 5,02 | 2,46 |
| P31947 | 14-3-3 protein sigma | # | n/a | 0,95 | n/a |
| P32856 | Syntaxin-2 | # | 0,31 | n/a | n/a |
| P35080 | Profilin-2 | # | 0,99 | n/a | n/a |
| P35232 | Prohibitin | # | n/a | 0,68 | n/a |
| P35579 | Myosin-9 | # | n/a | 1,25 | n/a |
| P36543 | V-type proton ATPase subunit E 1 | # | 2,09 | 1,56 | 0,74 |
| P36952 | Serpin B5 | # | n/a | 1,44 | n/a |
| P37840 | Alpha-synuclein | # | 0,37 | 0,92 | 2,45 |
| P38159 | RNA-binding motif protein, X chromosome | # | n/a | 1,11 | n/a |
| P38646 | Stress-70 protein, mitochondrial | # | 0,62 | 0,67 | 1,08 |
| P39023 | 60S ribosomal protein L3 | # | 0,42 | 0,53 | 1,25 |
| P39060 | Collagen alpha-1(XVIII) chain | # | n/a | 0,21 | n/a |
| P40121 | Macrophage-capping protein | # | n/a | 0,52 | n/a |
| P40926 | Malate dehydrogenase, mitochondrial | # | 0,56 | 0,71 | 1,27 |
| P43004 | Excitatory amino acid transporter 2 | # | n/a | 2,72 | n/a |
| P43243 | Matrin-3 | # | 0,25 | 1,02 | 4,01 |
| P45880 | Voltage-dependent anion-selective channel protein 2 | # | n/a | 1,83 | n/a |
| P45974 | Ubiquitin carboxyl-terminal hydrolase 5 | # | 1,33 | n/a | n/a |
| P46779 | 60S ribosomal protein L28 | # | n/a | 0,55 | n/a |
| P46781 | 40S ribosomal protein S9 | # | 0,56 | n/a | n/a |
| P46821 | Microtubule-associated protein 1B | # | 0,99 | 1,08 | 1,10 |
| P47914 | 60S ribosomal protein L29 | # | n/a | 0,69 | n/a |
| P47929 | Galectin-7 | # | n/a | 0,66 | n/a |
| P48047 | ATP synthase subunit O, mitochondrial | # | n/a | 0,93 | n/a |
| P48735 | Isocitrate dehydrogenase [NADP], mitochondrial | # | n/a | 1,67 | n/a |
| P49189 | 4-trimethylaminobutyraldehyde dehydrogenase | # | 0,64 | 0,59 | 0,92 |
| P49207 | 60S ribosomal protein L34 | # | 0,19 | n/a | n/a |
| P49327 | Fatty acid synthase | # | n/a | 0,59 | n/a |
| P49411 | Elongation factor Tu, mitochondrial | # | n/a | 0,83 | n/a |
| P49418 | Amphiphysin | # | 0,55 | 4,47 | 8,15 |
| P50395 | Rab GDP dissociation inhibitor beta | # | n/a | 0,39 | n/a |
| P50993 | Sodium/potassium-transporting ATPase subunit alpha-2 | # | 1,57 | 2,89 | 1,84 |
| P51149 | Ras-related protein Rab-7a | # | n/a | 0,50 | n/a |
| P51153 | Ras-related protein Rab-13 | # | n/a | 0,80 | n/a |
| P51991 | Heterogeneous nuclear ribonucleoprotein A3 | # | n/a | 1,00 | n/a |
| P52272 | Heterogeneous nuclear ribonucleoprotein M | # | 1,15 | 1,55 | 1,35 |
| P52564 | Dual specificity mitogen-activated protein kinase kinase 6 | # | n/a | 0,28 | n/a |
| P53621 | Coatomer subunit alpha | # | n/a | 1,04 | n/a |
| P53680 | AP-2 complex subunit sigma | # | 0,61 | n/a | n/a |
| P54136 | Arginine--tRNA ligase, cytoplasmic | # | n/a | 0,80 | n/a |
| P54289 | Voltage-dependent calcium channel subunit alpha-2/delta-1 | # | n/a | 2,73 | n/a |
| P55795 | Heterogeneous nuclear ribonucleoprotein H2 | # | n/a | 2,43 | n/a |
| P55809 | Succinyl-CoA:3-ketoacid coenzyme A transferase 1, mitochondrial | # | 1,48 | 0,98 | 0,67 |
| P56385 | ATP synthase subunit e, mitochondrial | # | n/a | 1,23 | n/a |
| P58107 | Epiplakin | # | n/a | 1,64 | n/a |
| P59998 | Actin-related protein 2/3 complex subunit 4 | # | n/a | 1,43 | n/a |
| P60174 | Triosephosphate isomerase | # | 0,69 | 1,02 | 1,49 |
| P60842 | Eukaryotic initiation factor 4A-I | # | n/a | 0,57 | n/a |
| P61088 | Ubiquitin-conjugating enzyme E2 N | # | n/a | 0,68 | n/a |
| P61106 | Ras-related protein Rab-14 | # | 0,47 | n/a | n/a |
| P61160 | Actin-related protein 2 | # | 1,00 | n/a | n/a |
| P61224 | Ras-related protein Rap-1b | # | 0,79 | n/a | n/a |
| P61247 | 40S ribosomal protein S3a | # | n/a | 0,56 | n/a |
| P61266 | Syntaxin-1B | # | n/a | 0,51 | n/a |
| P61313 | 60S ribosomal protein L15 | # | n/a | 0,41 | n/a |
| P61421 | V-type proton ATPase subunit d 1 | # | 1,26 | 1,20 | 0,95 |
| P61604 | 10 kDa heat shock protein, mitochondrial | # | 0,28 | 0,27 | 0,95 |
| P61626 | Lysozyme C | # | 0,39 | n/a | n/a |
| P61978 | Heterogeneous nuclear ribonucleoprotein K | # | 0,26 | 0,49 | 1,87 |
| P62191 | 26S protease regulatory subunit 4 | # | n/a | 0,94 | n/a |
| P62258 | 14-3-3 protein epsilon | # | 1,01 | 1,51 | 1,49 |
| P62424 | 60S ribosomal protein L7a | # | n/a | 0,48 | n/a |
| P62701 | 40S ribosomal protein S4, X isoform | # | 0,39 | n/a | n/a |
| P62714 | Serine/threonine-protein phosphatase 2A catalytic subunit beta isoform | # | 1,35 | n/a | n/a |
| P62760 | Visinin-like protein 1 | # | n/a | 1,61 | n/a |
| P62805 | Histone H4 | # | 1,11 | 1,30 | 1,17 |
| P62820 | Ras-related protein Rab-1A | # | 1,01 | n/a | n/a |
| P62826 | GTP-binding nuclear protein Ran | # | n/a | 0,51 | n/a |
| P62873 | Guanine nucleotide-binding protein G(I)/G(S)/G(T) subunit beta-1 | # | n/a | 1,01 | n/a |
| P62910 | 60S ribosomal protein L32 | # | n/a | 1,01 | n/a |
| P62937 | Peptidyl-prolyl cis-trans isomerase A | # | 3,70 | 0,89 | 0,24 |
| P63027 | Vesicle-associated membrane protein 2 | # | 2,22 | 1,16 | 0,52 |
| P63244 | Guanine nucleotide-binding protein subunit beta-2-like 1 | # | n/a | 0,88 | n/a |
| P67936 | Tropomyosin alpha-4 chain | # | 1,58 | n/a | n/a |
| P68371 | Tubulin beta-4B chain | # | 1,04 | 1,38 | 1,32 |
| P68871 | Hemoglobin subunit beta | # | 2,06 | 4,49 | 2,19 |
| P78324 | Tyrosine-protein phosphatase non-receptor type substrate 1 | # | 0,87 | n/a | n/a |
| P78352 | Disks large homolog 4 | # | 1,71 | n/a | n/a |
| P78371 | T-complex protein 1 subunit beta | # | n/a | 1,09 | n/a |
| P78559 | Microtubule-associated protein 1A | # | 0,48 | 1,05 | 2,21 |
| P81605 | Dermcidin | # | 0,39 | 0,65 | 1,68 |
| P83731 | 60S ribosomal protein L24 | # | n/a | 0,70 | n/a |
| P84098 | 60S ribosomal protein L19 | # | 0,56 | 0,56 | 0,99 |
| P84103 | Serine/arginine-rich splicing factor 3 | # | n/a | 1,48 | n/a |
| Q00325 | Phosphate carrier protein, mitochondrial | # | n/a | 1,17 | n/a |
| Q00577 | Transcriptional activator protein Pur-alpha | # | 0,24 | 1,87 | 7,78 |
| Q00610 | Clathrin heavy chain 1 | # | 0,74 | 1,34 | 1,82 |
| Q00839 | Heterogeneous nuclear ribonucleoprotein U | # | 0,72 | 0,98 | 1,36 |
| Q01130 | Serine/arginine-rich splicing factor 2 | # | n/a | 2,07 | n/a |
| Q01469 | Fatty acid-binding protein, epidermal | # | n/a | 0,52 | n/a |
| Q01484 | Ankyrin-2 | # | n/a | 1,19 | n/a |
| Q01813 | 6-phosphofructokinase type C | # | n/a | 0,81 | n/a |
| Q02252 | Methylmalonate-semialdehyde dehydrogenase [acylating], mitochondrial | # | n/a | 1,27 | n/a |
| Q02413 | Desmoglein-1 | # | 0,30 | 1,60 | 5,38 |
| Q02487 | Desmocollin-2 | # | n/a | 0,33 | n/a |
| Q02750 | Dual specificity mitogen-activated protein kinase kinase 1 | # | n/a | 1,51 | n/a |
| Q02978 | Mitochondrial 2-oxoglutarate/malate carrier protein | # | n/a | 0,96 | n/a |
| Q03252 | Lamin-B2 | # | 0,83 | 0,51 | 0,61 |
| Q04695 | Keratin, type I cytoskeletal 17 | # | 0,73 | 1,20 | 1,64 |
| Q04837 | Single-stranded DNA-binding protein, mitochondrial | # | 0,25 | 1,41 | 5,59 |
| Q04917 | 14-3-3 protein eta | # | 0,65 | 1,77 | 2,73 |
| Q05639 | Elongation factor 1-alpha 2 | # | 1,27 | n/a | n/a |
| Q06830 | Peroxiredoxin-1 | # | 2,28 | 1,42 | 0,62 |
| Q07020 | 60S ribosomal protein L18 | # | n/a | 0,41 | n/a |
| Q07955 | Serine/arginine-rich splicing factor 1 | # | n/a | 0,36 | n/a |
| Q08188 | Protein-glutamine gamma-glutamyltransferase E | # | n/a | 1,17 | n/a |
| Q08211 | ATP-dependent RNA helicase A | # | 0,11 | 0,65 | 5,69 |
| Q08554 | Desmocollin-1 | # | 1,54 | 1,08 | 0,70 |
| Q08722 | Leukocyte surface antigen CD47 | # | n/a | 0,91 | n/a |
| Q10567 | AP-1 complex subunit beta-1 | # | n/a | 1,21 | n/a |
| Q12860 | Contactin-1 | # | 1,64 | 0,68 | 0,42 |
| Q13449 | Limbic system-associated membrane protein | # | 0,99 | 1,14 | 1,15 |
| Q13509 | Tubulin beta-3 chain | # | n/a | 2,26 | n/a |
| Q13554 | Calcium/calmodulin-dependent protein kinase type II subunit beta | # | 1,07 | n/a | n/a |
| Q13765 | Nascent polypeptide-associated complex subunit alpha | # | n/a | 0,79 | n/a |
| Q13835 | Plakophilin-1 | # | n/a | 0,46 | n/a |
| Q14103 | Heterogeneous nuclear ribonucleoprotein D0 | # | 0,52 | n/a | n/a |
| Q14194 | Dihydropyrimidinase-related protein 1 | # | 0,70 | 0,62 | 0,89 |
| Q14204 | Cytoplasmic dynein 1 heavy chain 1 | # | n/a | 0,83 | n/a |
| Q14574 | Desmocollin-3 | # | n/a | 1,17 | n/a |
| Q14576 | ELAV-like protein 3 | # | n/a | 0,38 | n/a |
| Q14697 | Neutral alpha-glucosidase AB | # | n/a | 3,51 | n/a |
| Q14CN4 | Keratin, type II cytoskeletal 72 | # | n/a | 1,52 | n/a |
| Q15084 | Protein disulfide-isomerase A6 | # | n/a | 0,90 | n/a |
| Q15121 | Astrocytic phosphoprotein PEA-15 | # | 1,27 | n/a | n/a |
| Q15149 | Plectin | # | 0,19 | 0,92 | 5,00 |
| Q15366 | Poly(rC)-binding protein 2 | # | 0,77 | n/a | n/a |
| Q15517 | Corneodesmosin | # | n/a | 1,46 | n/a |
| Q15700 | Disks large homolog 2 | # | 1,32 | n/a | n/a |
| Q15836 | Vesicle-associated membrane protein 3 | # | n/a | 2,84 | n/a |
| Q16352 | Alpha-internexin | # | 1,38 | 0,83 | 0,60 |
| Q16629 | Serine/arginine-rich splicing factor 7 | # | n/a | 2,57 | n/a |
| Q16658 | Fascin | # | n/a | 0,89 | n/a |
| Q16836 | Hydroxyacyl-coenzyme A dehydrogenase, mitochondrial | # | n/a | 0,81 | n/a |
| Q53GQ0 | Estradiol 17-beta-dehydrogenase 12 | # | n/a | 0,59 | n/a |
| Q53RT3 | Retroviral-like aspartic protease 1 | # | n/a | 0,25 | n/a |
| Q5D862 | Filaggrin-2 | # | n/a | 1,82 | n/a |
| Q5TFQ8 | Signal-regulatory protein beta-1 isoform 3 | # | n/a | 0,64 | n/a |
| Q5VVQ6 | Ubiquitin thioesterase OTU1 | # | n/a | 0,61 | n/a |
| Q6KB66 | Keratin, type II cytoskeletal 80 | # | n/a | 0,43 | n/a |
| Q6PCE3 | Glucose 1,6-bisphosphate synthase | # | n/a | 0,34 | n/a |
| Q6UWP8 | Suprabasin | # | n/a | 0,72 | n/a |
| Q6ZN28 | Metastasis-associated in colon cancer protein 1 | # | 0,75 | n/a | n/a |
| Q6ZTR7 | Protein FAM92B | # | n/a | 1,34 | n/a |
| Q6ZVX7 | F-box only protein 50 | # | n/a | 0,56 | n/a |
| Q7L099 | Protein RUFY3 | # | 0,93 | n/a | n/a |
| Q7Z6G8 | Ankyrin repeat and sterile alpha motif domain-containing protein 1B | # | n/a | 1,22 | n/a |
| Q7Z7L9 | Zinc finger and SCAN domain-containing protein 2 | # | n/a | 0,41 | n/a |
| Q86VP6 | Cullin-associated NEDD8-dissociated protein 1 | # | 0,50 | n/a | n/a |
| Q86YZ3 | Hornerin | # | n/a | 1,20 | n/a |
| Q8IYB4 | PEX5-related protein | # | n/a | 0,64 | n/a |
| Q8IYT4 | Katanin p60 ATPase-containing subunit A-like 2 | # | 0,56 | n/a | n/a |
| Q8N1N4 | Keratin, type II cytoskeletal 78 | # | n/a | 0,64 | n/a |
| Q8N573 | Oxidation resistance protein 1 | # | n/a | 6,87 | n/a |
| Q8NCB2 | CaM kinase-like vesicle-associated protein | # | 1,46 | n/a | n/a |
| Q8TF72 | Protein Shroom3 | # | 0,35 | 0,35 | 1,00 |
| Q8WVK7 | Spindle and kinetochore-associated protein 2 | # | n/a | 0,56 | n/a |
| Q8WVV4 | Protein POF1B | # | n/a | 1,12 | n/a |
| Q8WXF1 | Paraspeckle component 1 | # | n/a | 1,00 | n/a |
| Q92499 | ATP-dependent RNA helicase DDX1 | # | n/a | 0,92 | n/a |
| Q92561 | Phytanoyl-CoA hydroxylase-interacting protein | # | n/a | 0,55 | n/a |
| Q92598 | Heat shock protein 105 kDa | # | 2,36 | 0,46 | 0,19 |
| Q92599 | Septin-8 | # | n/a | 1,36 | n/a |
| Q92686 | Neurogranin | # | n/a | 0,78 | n/a |
| Q92752 | Tenascin-R | # | 1,72 | 1,41 | 0,82 |
| Q92777 | Synapsin-2 | # | n/a | 3,35 | n/a |
| Q93050 | V-type proton ATPase 116 kDa subunit a isoform 1 | # | 1,24 | n/a | n/a |
| Q969P0 | Immunoglobulin superfamily member 8 | # | 0,65 | n/a | n/a |
| Q969V3 | Nicalin | # | n/a | 3,78 | n/a |
| Q96CW1 | AP-2 complex subunit mu | # | 0,65 | 1,30 | 2,01 |
| Q96F07 | Cytoplasmic FMR1-interacting protein 2 | # | n/a | 0,65 | n/a |
| Q96GD0 | Pyridoxal phosphate phosphatase | # | 1,34 | n/a | n/a |
| Q96GW7 | Brevican core protein | # | 2,20 | n/a | n/a |
| Q96KP4 | Cytosolic non-specific dipeptidase | # | n/a | 1,52 | n/a |
| Q96P63 | Serpin B12 | # | n/a | 1,21 | n/a |
| Q96QA5 | Gasdermin-A | # | n/a | 0,49 | n/a |
| Q99460 | 26S proteasome non-ATPase regulatory subunit 1 | # | n/a | 0,55 | n/a |
| Q99536 | Synaptic vesicle membrane protein VAT-1 homolog | # | n/a | 1,03 | n/a |
| Q99623 | Prohibitin-2 | # | 0,97 | n/a | n/a |
| Q99798 | Aconitate hydratase, mitochondrial | # | 1,04 | 0,81 | 0,78 |
| Q99962 | Endophilin-A1 | # | 1,47 | 0,58 | 0,39 |
| Q9BPW8 | Protein NipSnap homolog 1 | # | 0,93 | n/a | n/a |
| Q9BQE3 | Tubulin alpha-1C chain | # | 1,22 | 0,94 | 0,77 |
| Q9BVC6 | Transmembrane protein 109 | # | n/a | 0,28 | n/a |
| Q9BY11 | Protein kinase C and casein kinase substrate in neurons protein 1 | # | 1,81 | 1,65 | 0,91 |
| Q9H0E2 | Toll-interacting protein | # | 0,71 | n/a | n/a |
| Q9H115 | Beta-soluble NSF attachment protein | # | 1,73 | 0,87 | 0,50 |
| Q9H1K4 | Mitochondrial glutamate carrier 2 | # | 1,22 | 0,87 | 0,71 |
| Q9H492 | Microtubule-associated proteins 1A/1B light chain 3A | # | n/a | 1,72 | n/a |
| Q9NQW7 | Xaa-Pro aminopeptidase 1 | # | n/a | 1,24 | n/a |
| Q9NRW1 | Ras-related protein Rab-6B | # | 0,18 | n/a | n/a |
| Q9NSD9 | Phenylalanine--tRNA ligase beta subunit | # | n/a | 0,65 | n/a |
| Q9NTU7 | Cerebellin-4 | # | 0,09 | n/a | n/a |
| Q9NVJ2 | ADP-ribosylation factor-like protein 8B | # | n/a | 0,42 | n/a |
| Q9NYC9 | Dynein heavy chain 9, axonemal | # | n/a | 0,58 | n/a |
| Q9NZH8 | Interleukin-36 gamma | # | n/a | 1,46 | n/a |
| Q9P121 | Neurotrimin | # | 0,38 | n/a | n/a |
| Q9P2K5 | Myelin expression factor 2 | # | n/a | 3,55 | n/a |
| Q9P2R7 | Succinyl-CoA ligase [ADP-forming] subunit beta, mitochondrial | # | 0,84 | 1,12 | 1,33 |
| Q9UBB6 | Neurochondrin | # | n/a | 0,72 | n/a |
| Q9UI12 | V-type proton ATPase subunit H | # | 1,35 | n/a | n/a |
| Q9ULU8 | Calcium-dependent secretion activator 1 | # | 0,87 | n/a | n/a |
| Q9UMF0 | Intercellular adhesion molecule 5 | # | 0,85 | n/a | n/a |
| Q9UN86 | Ras GTPase-activating protein-binding protein 2 | # | n/a | 1,14 | n/a |
| Q9UPY8 | Microtubule-associated protein RP/EB family member 3 | # | n/a | 0,60 | n/a |
| Q9Y285 | Phenylalanine--tRNA ligase alpha subunit | # | 0,53 | 0,80 | 1,52 |
| Q9Y2A7 | Nck-associated protein 1 | # | 0,64 | n/a | n/a |
| Q9Y2J2 | Band 4.1-like protein 3 | # | n/a | 0,66 | n/a |
| Q9Y3I0 | tRNA-splicing ligase RtcB homolog | # | n/a | 0,56 | n/a |
| Q9Y4L1 | Hypoxia up-regulated protein 1 | # | n/a | 0,91 | n/a |
| Q9Y5K8 | V-type proton ATPase subunit D | # | n/a | 0,36 | n/a |
| Q9Y639 | Neuroplastin | # | n/a | 0,26 | n/a |
| Q9Y6C2 | EMILIN-1 | # | n/a | 0,39 | n/a |
